# TOM1L1 drives membrane delivery of MT1-MMP to promote ERBB2-induced breast cancer cell invasion

**DOI:** 10.1038/ncomms10765

**Published:** 2016-02-22

**Authors:** Clément Chevalier, Guillaume Collin, Simon Descamps, Heiani Touaitahuata, Valérie Simon, Nicolas Reymond, Laurent Fernandez, Pierre-Emmanuel Milhiet, Virginie Georget, Serge Urbach, Laurence Lasorsa, Béatrice Orsetti, Florence Boissière-Michot, Evelyne Lopez-Crapez, Charles Theillet, Serge Roche, Christine Benistant

**Affiliations:** 1 Montpellier University, Centre de Recherche de Biochimie Macromoléculaire, CNRS UMR 5237, 34293 Montpellier, France; 2 Centre de Biochimie Structurale, CNRS UMR 5048-INSERM UMR 1054, 29 rue de navacelles, 34090 Montpellier, France; 3 Montpellier RIO Imaging Facility, 34293 Montpellier, France; 4 Functional Proteomics Platform, 34090 Montpellier, France; 5 IRCM, Institut de Recherche en Cancérologie de Montpellier; INSERM U896, 34298 Montpellier, France; 6 Translational Research Unit, Institut régional du Cancer de Montpellier (ICM)-Val d'Aurelle, 34298 Montpellier, France

## Abstract

ERBB2 overexpression in human breast cancer leads to invasive carcinoma but the mechanism is not clearly understood. Here we report that *TOM1L1* is co-amplified with *ERBB2* and defines a subgroup of *HER2*^*+*^*/ER*^*+*^ tumours with early metastatic relapse. *TOM1L1* encodes a GAT domain-containing trafficking protein and is a SRC substrate that negatively regulates tyrosine kinase signalling. We demonstrate that TOM1L1 upregulation enhances the invasiveness of ERBB2-transformed cells. This pro-tumoural function does not involve SRC, but implicates membrane-bound membrane-type 1 MMP (MT1-MMP)-dependent activation of invadopodia, membrane protrusions specialized in extracellular matrix degradation. Mechanistically, ERBB2 elicits the indirect phosphorylation of TOM1L1 on Ser321. The phosphorylation event promotes GAT-dependent association of TOM1L1 with the sorting protein TOLLIP and trafficking of the metalloprotease MT1-MMP from endocytic compartments to invadopodia for tumour cell invasion. Collectively, these results show that TOM1L1 is an important element of an ERBB2-driven proteolytic invasive programme and that *TOM1L1* amplification potentially enhances the metastatic progression of ERBB2-positive breast cancers.

## Abstract (web summary)

ERBB2 overexpression in human breast cancer leads to invasion and metastasis. Here the authors report that ERBB2 induces indirect phosphorylation of TOM1L1 that promotes trafficking of the metalloprotease MT1-MMP to invadopodia, which leads to tumour cell invasion.

## Graphical abstract

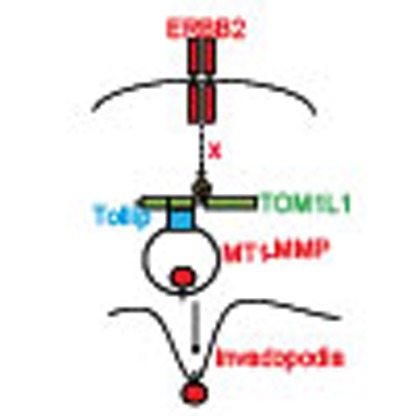

Genetic and epigenetic alterations in breast cancer cells eventually result in invasive carcinoma. The *ERBB2* oncogene (also known as HER2 or neu), which encodes a tyrosine kinase receptor of the EGFR family, is amplified and overexpressed in about 20% of breast tumours. Overexpressed ERBB2 is abnormally concentrated at the plasma membrane, promoting receptor homo-dimerization or hetero-dimerization with additional members of the EGFR family. Dimerized receptors display strong kinase activity and induce oncogenic signalling, leading to malignant cell transformation^1^. ERBB2 oncogenic potential and cell surface availability have led to the development of targeted anti-ERBB2 antibodies, such as trastuzumab (Herceptin) that has become the standard care for patients with ERBB2-positive breast cancer. However, 50% of these patients respond poorly and/or develop tumour resistance or show strong adverse side effects. Therefore, there is an urgent need to better understand the molecular basis of ERBB2-induced metastatic malignancy for developing new targeted treatments.

The mechanism by which abnormal ERBB2 expression leads to metastatic progression is only partially elucidated. Several components of the invasive programme driven by ERBB2 have been identified and include the transmembrane proteins Integrin beta 4 (ref. 2) and PlexinB1 (ref. 3), small GTPases of the Rho family^4^,^5^, microtubules, ACF7 and Memo^6^,^7^, miR-21 (ref. 8) and the protein kinase HUNK^9^. ERBB2-mediated invasion is also strongly coupled to the capacity of tumour cells to induce extracellular matrix (ECM) proteolysis through the activation of urokinase plasminogen activator, lysosomal cathepsins and multi-domain zinc-dependent endopeptidases or matrix metalloproteases (MMPs)^10^,^11^,^12^. Interestingly, membrane-bound membrane-type 1 MMP (MT1-MMP) has emerged as a crucial inducer of tissue invasion and is involved in the rupturing of basement membranes by tumour cells and also in cell invasion through interstitial tissues rich in type-I collagen^13^. MT1-MMP invasive function is tightly controlled through intracellular trafficking and catalytic activity^14^. For instance, MT1-MMP is activated by proteolytic cleavage in the trans-Golgi network and partitioned in specialized membrane domains called invadopodia. These F-actin-enriched structures secrete proteases at cell-ECM contact sites for matrix degradation and cell invasion^15^,^16^. Yet, the role of MT1-MMP and invadopodia activity in ERBB2-induced cell invasion is largely unknown.

Target of MYB1-like protein 1 (TOM1L1, also known as Srcasm) has been recently identified as a gene relevant to bone metastasis in breast cancer^17^ and we observed that *TOM1L1,* which is located on chromosome 17q22, is frequently co-amplified with *ERBB2* in breast cancer^18^, suggesting that TOM1L1 could have a pro-oncogenic function. TOM1L1 is an adaptor protein of the TOM1 family with post-Golgi trafficking and signalling functions. TOM1, TOM1L1 and TOM1L2 proteins include a VHS (Vps27/Hrs/Stam) domain, like Hrs, Stam1, Stam2, GGA1, GGA2 and GGA3. In addition, GGA and TOM1 contain an evolutionary conserved GAT (GGA and TOM1) domain also involved in trafficking functions. The VHS and GAT domains of GGAs mediate protein trafficking between the trans-Golgi network and endosomes through binding to transmembrane cargos and to the small GTPase ADP-ribosylation factor, respectively. On the other hand, the VHS and GAT domains of TOM1 family members^19^ bind to endosomal sorting proteins, such as TOLLIP (Toll-interacting protein) or ubiquitin in a mutually exclusive manner^20^,^21^. TOM1L1 comprises a unique C terminus with several protein interaction motifs, including a SRC-SH3-binding site, a leucine-rich motif with binding affinity for clathrin heavy chain (CHC) and three tyrosine residues that, when phosphorylated by SRC, create binding sites for the SH2-containing signalling proteins GRB2, p85 and members of the SRC family^22^,^23^. TOM1L1 participates in EGFR endocytosis for lysosomal degradation through a SRC-dependent and CHC-dependent mechanism^24^. It also associates with CHC to regulate SRC membrane partitioning required for SRC mitogenic and transforming activity^25^. In contrast with the observations in human breast cancer, in transgenic mice, Srcasm inhibits Fyn-induced neoplasia via kinase downregulation and modulation of p53 and Notch. All this data support a model in which TOM1L1 engages a SRC-like-dependent mechanism to control tyrosine kinase signalling^22^,^23^,^24^,^25^.

Here we addressed the contribution of *TOM1L1-ERBB2* co-amplification in ERBB2 oncogenic signalling. We show that their co-amplification is associated with worse prognosis in patients with oestrogen receptor-positive (ER+) breast cancer. We next demonstrate that TOM1L1 enhances ERBB2-induced cell invasiveness by promoting invadopodia formation and MT1-MMP trafficking to the plasma membrane. This process is independent from SRC activity, but requires TOM1L1 phosphorylation on Ser321 and association with TOLLIP. Thus, TOM1L1 is an important element of an ERBB2-driven proteolytic invasive programme and *TOM1L1* amplification potentially enhances the metastatic progression of ERBB2-positive breast cancers.

## Results

### TOM1L1 is co-expressed with ERBB2 in breast cancer

**Figure 1:**
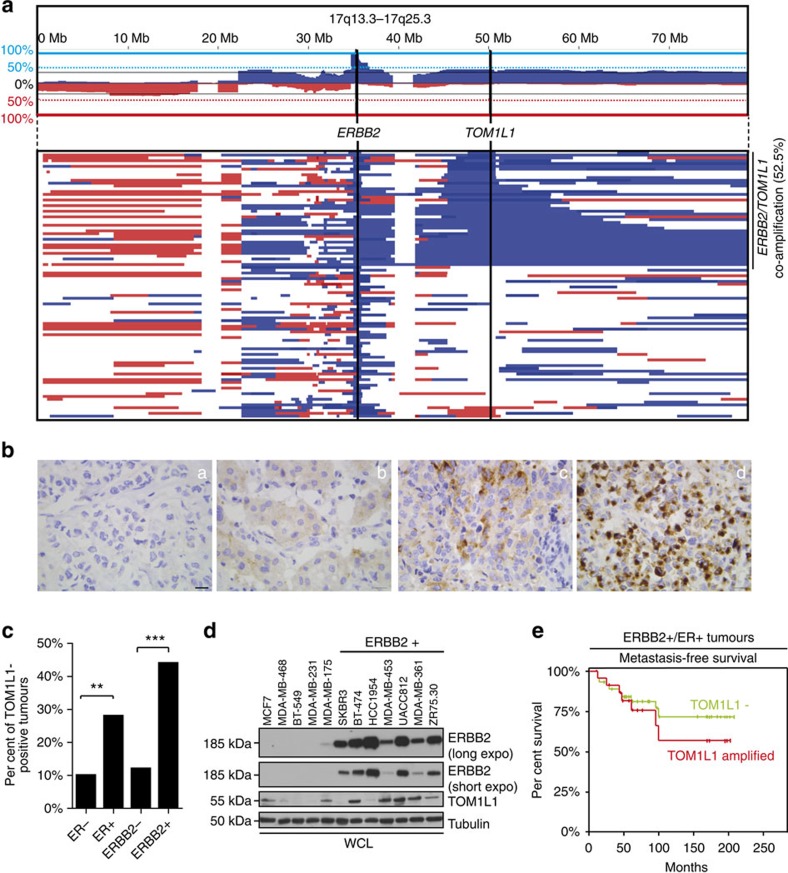
TOM1L1 is co-expressed with ERBB2 in breast cancer. (**a**) The *ERBB2* and *TOM1L1* loci are frequently co-amplified in breast cancer. Upper part: cumulative array-CGH copy number frequency plots of chromosome 17 in breast tumours with *ERBB2* amplification. Copy number gains are in blue, losses in red. Tumours with a log2 ratio higher than the threshold of 0.25 were considered as amplified. Lower part: copy number profiles at chromosome 17 in individual tumours (each line is one tumour). The colour code is the same as in the upper part. About 52.5% of ERBB2-positive tumours show *TOM1L1* co-amplification. (**b**) Representative images showing TOM1L1 protein expression in breast cancer samples with absence (A), weak (B), moderate (C) and strong (D) immunoreactivity. Scale bar, 10 μm. (**c**) TOM1L1 expression was significantly associated with ER and ERBB2 expression. Histograms shows the results of screening by IHC of TOM1L1 expression in 108 breast tumours encompassing the four major subtypes (ER−, ER+, ERBB2− and ERBB2+). Statistical analysis was done with Student's *t*-test on raw data. **P<0.01 and ***P<0.001. (**d**) TOM1L1 is overexpressed in ERBB2+ cell lines. Western blot analyses of TOM1L1, ERBB2 and tubulin (used as loading control) expression in 12 breast cancer cell lines. (**e**) Kaplan–Meier survival analysis of patients with ERBB2+/ER+ breast cancer from the database of ref. 27. Patients were stratified based on *TOM1L1* amplification (red curve) and were compared with patients with no amplification (green curve). The log-rank test was used to determine the statistical significance (*P* value≤0.05).

We first confirmed that in breast cancer, the *ERBB2* locus at 17q12 (37.85 Mb) is frequently co-amplified with loci located at 17q22 (53 Mb) and 17q23 (Fig. 1a)^18^. Analysis of a set of 402 breast tumours showed that *TOM1L1* was the most frequently co-amplified gene with *ERBB2* in the 17q22-q23 region (Supplementary Table 1). *TOM1L1 g*ene amplification was associated with TOM1L1 transcript upregulation (Supplementary Table 2). Moreover, in breast cancer samples, TOM1L1 protein expression, assessed by immunohistochemistry (IHC) (Fig. 1b), was significantly associated with both ER (*P*=0.027) and ERBB2 (*P*=0.001) expression (Fig. 1c and Supplementary Table 3). Similarly, TOM1L1 protein level evaluated by western blotting was high in five out of seven ERBB2+ cell lines, whereas it was absent or lower in the five ERBB2-negative cell lines we tested (Fig. 1d). Finally, metastasis-free survival was reduced in patients with ERBB2+/ER+ breast cancer in which *TOM1L1* was also amplified compared with those without *TOM1L1* amplification (patients selected from our previous work^26^) (Fig. 1e). These results suggest a cooperative effect of ERBB2 and TOM1L1 in ER+ breast tumours and a positive role for TOM1L1 in ERBB2-driven malignancy.

### TOM1L1 regulates ERBB2-induced cell invasion

**Figure 2:**
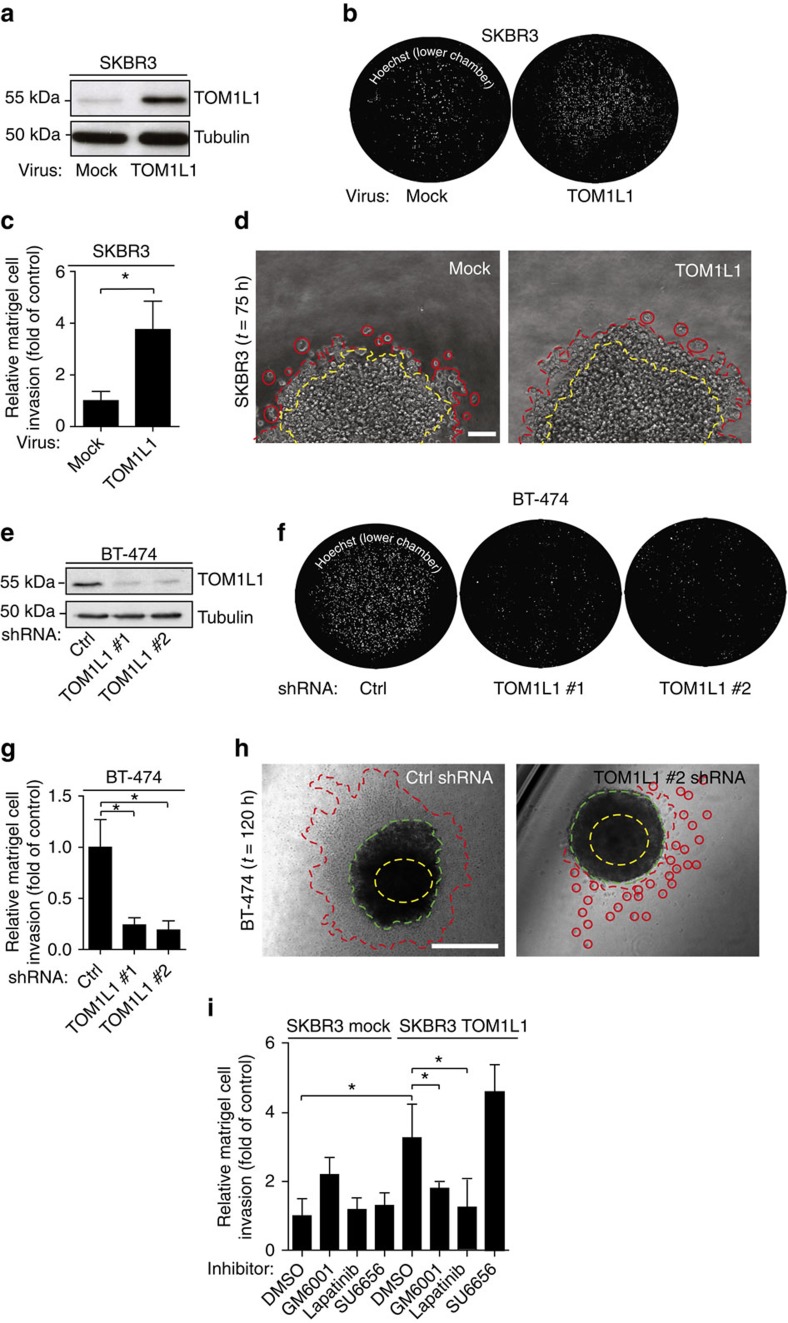
TOM1L1 regulates ERBB2-driven cell invasion. (**a**) TOM1L1 expression was assessed by western blotting in SKBR3 cells infected with empty retroviruses (mock) or coding TOM1L1. (**b**) SKBR3 cells infected as in **a** were seeded in the upper compartment of a Boyden chamber containing 1 mg ml^−1^ matrigel. After 48 h, cells present in the lower chamber were visualized by Hoechst staining. (**c**) Quantification of **b**. The histogram shows the invasion ratio normalized to control conditions (*n*=3). (**d**) SKBR3 cells infected as indicated were embedded as spheroids in a collagen and matrigel matrix for 75 h and then imaged by phase contrast microscopy. Yellow dotted line shows the sphere size at *t*=0 and the red dotted line shows invasive cells or invasive fronts at the end of the experiment. Invasive fronts were twice larger in TOM1L1-expressing cells than in controls cells (*n*=4). Scale bar, 100 μm. (**e**) TOM1L1 expression was assessed by western blotting in BT-474 cells infected with viruses containing control shRNA (Ctrl) or two different TOM1L1 shRNAs (TOM1L1 #1 and #2). (**f**) Invasion of the BT-474 infected cells was assessed in Boyden chambers with matrigel as in **b**. Cells in the lower chamber were stained with Hoechst and counted as in **c** (*n*=3) (**g**,**h**) Infected BT-474 cells were embedded as spheroids in a collagen and matrigel matrix for 5 days and imaged by phase contrast microscopy. Yellow dotted line shows sphere size at *t*=0, green dotted line shows sphere size at *t*=120 h and red dotted line shows invasive cells or invasive fronts. The surface explored by the cells was decreased by 1.5-fold in TOM1L1 silenced cells compared with controls (*n*=3). Scale bars, 400 μm. (**i**) SKBR3 cells infected as indicated were seeded in Boyden chambers as in **b**,**c** and incubated with DMSO, 12.5 μM GM6001 (MMP inhibitor), 1 μM lapatinb (an EGFR/ERBB2 inhibitor) and 2 μM SU6656 (SRC inhibitor) for 48 h. The histogram shows the invasion ratio normalized to control conditions (*n*=10). All histograms in Fig. 2 show mean±s.e.m. *P≤0.05 (Student's *t*-test).

**Figure 3:**
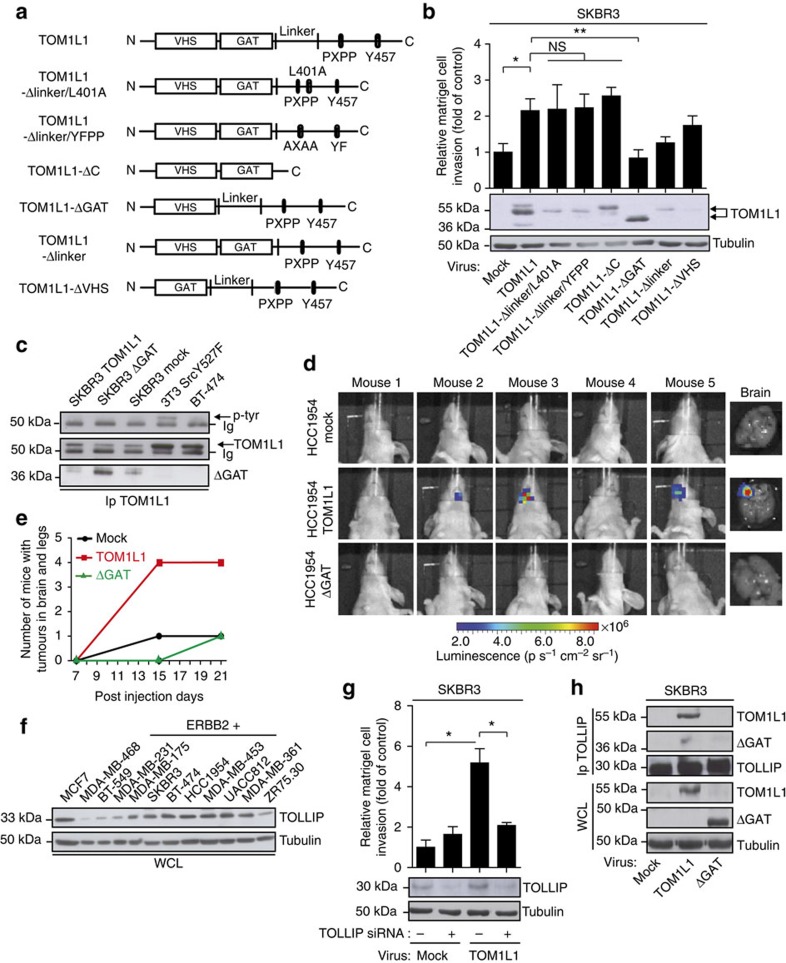
TOM1L1 pro-invasive activity requires ERBB2-induced interaction of its GAT domain with TOLLIP. (**a**) Schematic showing TOM1L1 and the different mutants used in the study. (**b**) Upper panel: invasion of SKBR3 cells infected with the indicated viruses was assessed in Boyden chambers with matrigel. The histogram shows the invasion ratio normalized to the control value after 48 h of invasion (*n*=4). Mean±s.e.m *P<0.05, **P<0.01, NS, no significant (Student's *t*-test). Lower panel: western blots showing the expression of the various TOM1L1 mutants in SKBR3 cells. (**c**) Lysates from BT-474, 3T3-SRCY527F and SKBR3 cell lines expressing the indicated constructs were immunoprecipitated with an anti-TOM1L1 antibody and blotted as indicated. Note that only the 3T3-SRCY527F cell lysate shows a band corresponding to p-tyr above the Ig band. (**d**,**e**) *In vivo* metastasis assay: luciferase-expressing HCC-1954 cells infected as indicated were injected in mice hearts (left ventricle). At the indicated times, mice were injected intraperitoneally with luciferine and imaged. (**d**) Mice imaging show higher bioluminescence signal in the brain of mice injected with TOM1L1-expressing cells compared with mock or ΔGAT-expressing cells (day 21 post injection). Right panel shows representative images of mice brains in each condition. (**e**) Quantification in time of the number of mice exhibiting bioluminescence signals in brain or legs. Note that bioluminescence signals are detected earlier and in more mice (day 15) when injected with cells that express TOM1L1 than with control cells (mock) or the ΔGAT mutant. (**f**) Western blot analysis of TOLLIP expression in 12 breast cancer cell lines. Note that TOLLIP is preferentially expressed in ERBB2+ cell lines. Lysates used were the same as in Fig. 1d. (**g**) Upper panel: invasion of SKBR3 cells infected as indicated and transfected twice with control (−) or *TOLLIP* siRNAs (+) was assessed and quantified as in **b** (*n*=5). Mean±s.e.m *P<0.05 (Student's *t*-test). Lower panel: the efficiency of TOLLIP silencing was confirmed by western blot analysis of TOLLIP expression in the transfected cells. (**h**) Whole cell lysates of SKBR3 cells infected with indicated viruses were immunoprecipitated with an anti-TOLLIP antibody and immunoblotted as shown.

We next investigated TOM1L1 role in breast cancer by manipulating its expression level in ERBB2+/ER+/TOM1L1− SKBR3 cells (by overexpression) and in ERBB2+/ER+/TOM1L1+ BT-474 cells (by short interfering RNA (shRNA)-mediated silencing). TOM1L1 overexpression in this last cell line is linked to its gene amplification (not shown). Modulation of TOM1L1 expression did not have any effect on growth and migration of these breast cancer cells (Supplementary Fig. 1a,b). In contrast, TOM1L1 overexpression in SKBR3 cells increased by four times cell invasion in Boyden chambers and twice in three-dimensional (3D) multicellular spheroid assays compared with mock-transfected cells (Fig. 2a–d). TOM1L1 depletion in BT-474 cells reduced by 4 times cell invasion in Boyden assays and by 1.5 times the surface explored by cells in 3D multicellular spheroid assays compared with cells expressing control shRNA (Fig. 2e–h). TOM1L1 pro-invasive function was associated with a change in migration modes: we observed less single cells and more collective fronts or strands in 3D when TOM1L1 was expressed, behaviour previously described to be protease dependant^27^. Accordingly, we found that TOM1L1-induced cell invasion was sensitive to proteases inhibition (Fig. 2i and Supplementary Fig. 2c). It was also associated with ERBB2 activity since Lapatinib, a dual EGFR/ERBB2 inhibitor that mainly acts through inhibition of ERBB2 in SKBR3 and BT-474 cells^28^, also strongly reduced this invasive activity. However, it was largely independent from SRC-like activities (Fig. 2i and Supplementary Fig. 2c). Similar data were obtained using a matrix of collagen 1 from rat tail with intact telopeptides (Supplementary Fig. 2a,b). We next confirmed this data by a structure–function analysis (Fig. 3a). TOM1L1 mutants that cannot bind to SRC (Δlinker/YFPP) or CHC (Δlinker/L401A)^25^ retained the full capacity to mediate cell invasion (Fig. 3b), suggesting that TOM1L1 uses SRC- and CHC-binding-independent mechanisms for ERBB2-induced cell invasion. Accordingly, TOM1L1 was not tyrosine-phosphorylated in ERBB2+ SKBR3 and BT-474 cells, differently from what was observed in SRC-transformed cells (Fig. 3c)^25^. Overall, our results suggest that TOM1L1 uses a mechanism independent of tyrosine phosphorylation, SRC and CHC binding to promote cell invasion.

### TOM1L1 activity needs interaction with TOLLIP

Deletion of the GAT domain (ΔGAT mutant) abolished TOM1L1 pro-invasive activity in SKBR3 cells (Fig. 3a,b) and in HCC-1954 cells (another ERBB2+/ER+/TOM1L1− breast cancer cell line) (Supplementary Fig. 2d,e). When injected in mammary fat pad of nude mice, none of these cells developed metastases, suggesting that these cells are poorly metastatic in our experimental setting (not shown). Moreover, TOM1L1 overexpression in HCC-1954 cells did not induce metastasis either, suggesting that TOM1L1 alone is not sufficient to promote metastatic progression. However, when injected in the left ventricle, HCC-1954 cells induced detectable metastatic nodules in recipient mice. Interestingly, we observed that HCC-1954 cells that overexpress TOM1L1 produced more rapidly metastases that reached the brain in more animals than mock-transfected HCC-1954 cells (mock) or ΔGAT-expressing HCC-1954 cells (Fig. 3d,e). These findings were confirmed using NIH-3T3 cells transformed with an oncogenic version of ERBB2 (3T3-neu cells) (Supplementary Fig. 3). As before, TOM1L1 increased *in vitro* and *in vivo* cell invasion in a GAT- and ERBB2-dependent manner, showing that TOM1L1 role in ERBB2 invasive signalling is conserved and validating this model for further studies (Supplementary Fig. 3). We then looked for factors that interact with TOM1L1-GAT domain and that could be involved in this process. The vesicular trafficking protein TOLLIP was an attractive candidate as it binds to TOM1L1-GAT domain^2^ and is upregulated in some breast cancers^29^ and mainly in ERBB2+ breast cancer cell lines (Fig. 3f). In support to this hypothesis, siRNA-mediated TOLLIP depletion inhibited TOM1L1 pro-invasive activity in SKBR3 cells that overexpress TOM1L1 compared with non-silenced cells (Fig. 3g and Supplementary Fig. 4a). TOM1L1 also co-immunoprecipitated with TOLLIP in lysates from SKBR3 cells that overexpress TOM1L1 and this interaction required the TOM1L1-GAT domain and ERBB2 activity (Fig. 3h and Supplementary Fig. 4b,c). Thus, ERBB2 induces cell invasion and metastasis by a novel mechanism that involves TOM1L1 interaction with TOLLIP through its GAT domain.

### TOM1L1 regulates invadopodia and MT1-MMP activity

**Figure 4:**
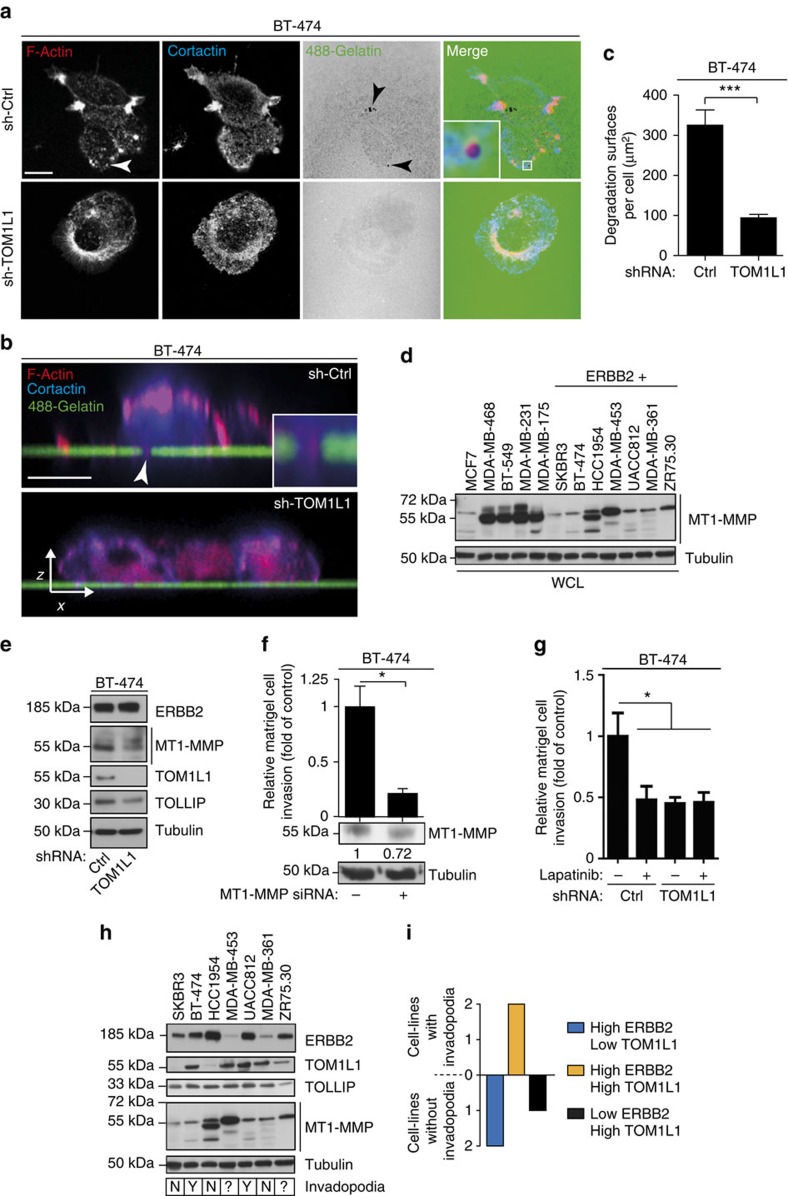
TOM1L1 regulates invadopodia and requires MT1-MMP. (**a**) BT-474 cells infected with the indicated shRNAs were plated onto Oregon Green 488 gelatin-coated coverslips. After 6–24 h, cells were fixed and labelled with the relevant antibodies to visualize F-actin and cortactin by confocal microscopy. Arrowheads point to actin punctiform structure (white) and degradation of the gelatin matrix (black). Inset shows higher magnification of the boxed region. Scale bar, 10 μm. (**b**) Same experiment as in **a** but cells were imaged using a confocal orthogonal (*x/z*) slice view. Inset shows higher magnification of the arrowhead-pointed zone. Scale bar, 10 μm. (**c**) Quantification of Oregon Green 488 gelatin degradation areas in μm^2^ (*n*=3). Mean±s.e.m., ***P<0.001 (Student's *t*-test). (**d**) Western blot showing MT1-MMP expression in 12 breast cancer cell lines. Tubulin is used as loading control. Lysates used were the same as in Figs 1d and 3f. (**e**) Indicated proteins expression was assessed by immunoblot in BT-474 cells lysates expressing indicated shRNAs. (**f**) Upper panel: 48 h after transfection of control or *MT1-MMP*-specific siRNAs, invasion of BT-474 cells was assessed in Boyden chambers with matrigel (*n*=5). Mean±s.e.m., *P≤0.05 (Student's *t*-test). Lower panel: WCL of BT-474 cells transfected with control (−) or *MT1-MMP* (+) siRNAs were immunoblotted to check MT1-MMP expression. Numbers shown under MT1-MMP represents quantification of MT1-MMP silencing compared with control condition. (**g**) Invasion of BT-474 cells expressing indicated shRNAs was assessed in Boyden chamber with matrigel in the presence of 2μM Lapatinib (+) or DMSO (−). (**h**) Western blot showing expression of the indicated proteins in seven ERBB2+ breast cancer cell lines. Blots are the same as those presented in Figs 1d, 3f and . Lower panel indicates the presence of invadopodia in the different cell lines. N=not detected, Y=yes, ?=not tested. (**i**) Correlation between ERBB2/TOM1L1 and invadopodia. Histogram indicates the number of cell lines exhibiting invadopodia in function of ERBB2 and TOM1L1 expression. High ERBB2/Low TOM1L1 cell lines are SKBR3 and HCC-1954. High ERBB2/High TOM1L1 cell lines are BT-474 and UACC812 and Low ERBB2/High TOM1L1 cell line is MDA-MB 361.

We next characterized the pro-invasive function of TOM1L1 in more detail. As invadopodia lie at the cross-road between cell traffic and invasion, we first investigated these membrane-enriched structures, which are characterized by the presence of F-actin- and cortactin, in ERBB2-transformed cell lines. BT-474 and 3T3-neu cells displayed invadopodia-like structures when plated on matrigel or gelatin (Fig. 4a–c and Supplementary Fig. 5a–e). The invadopodia nature of these structures was confirmed by *in situ* zymography that showed the overlapping of invadopodia-like structures with degradation holes in the fluorescent gelatin matrix (arrowheads in Fig. 4a,b). In addition, co-localization of MT1-MMP with invadopodia markers, such as p-421 cortactin, was also observed (Supplementary Fig. 5c). TOM1L1 silencing in BT-474 cells indicated that TOM1L1 regulated invadopodia formation (Fig. 4a,b and Supplementary Fig. 5a–e) and activity (Fig. 4a,c and Supplementary Fig. 5d,e). Consistent with a role in cell proteolytic activity, TOM1L1 also regulates MT1-MMP (expression shown in Fig. 4d) peptidic cleavage involved in its maturation (Fig. 4e and Supplementary Fig. 5f). The essential role of MT1-MMP in invadopodia activity was confirmed by the strong reduction of matrigel invasion and migration in collagen matrix on MT1-MMP silencing in BT-474 (Fig. 4f) and 3T3-neu cells (Supplementary Figs 3b and 5g,h). The essential role of ERBB2 activity on these invasive processes was confirmed using Lapatinib treatment (Fig. 4g and Supplementary Fig. 5a,b). Consistent with this data, we also found a correlation between the capacity of ERBB2+ tested tumour cell lines to induce invadopodia formation and a high expression level of ERBB2 and TOM1L1, (Fig. 4h,i). Finally, we checked whether this TOM1L1 invasive role was conserved in breast tumour cells by overexpressing TOM1L1 in the ERBB2- and TOM1L1-negative breast cancer cell line MDA-MB-231 (Supplementary Fig. 6). We found that TOM1L1 overexpression reduced both invasion and invadopodia activity of MDA MB-231 cells (Supplementary Fig. 6b–d), suggesting an opposite role of TOM1L1 on invasive activity of cells that do not express ERBB2 and whose invadopodia activity depends on Src kinases activities. Altogether, these data demonstrate that TOM1L1 regulates the formation of invadopodia of ERBB2+ transformed cells.

### TOM1L1 regulates MT1-MMP membrane localization

**Figure 5:**
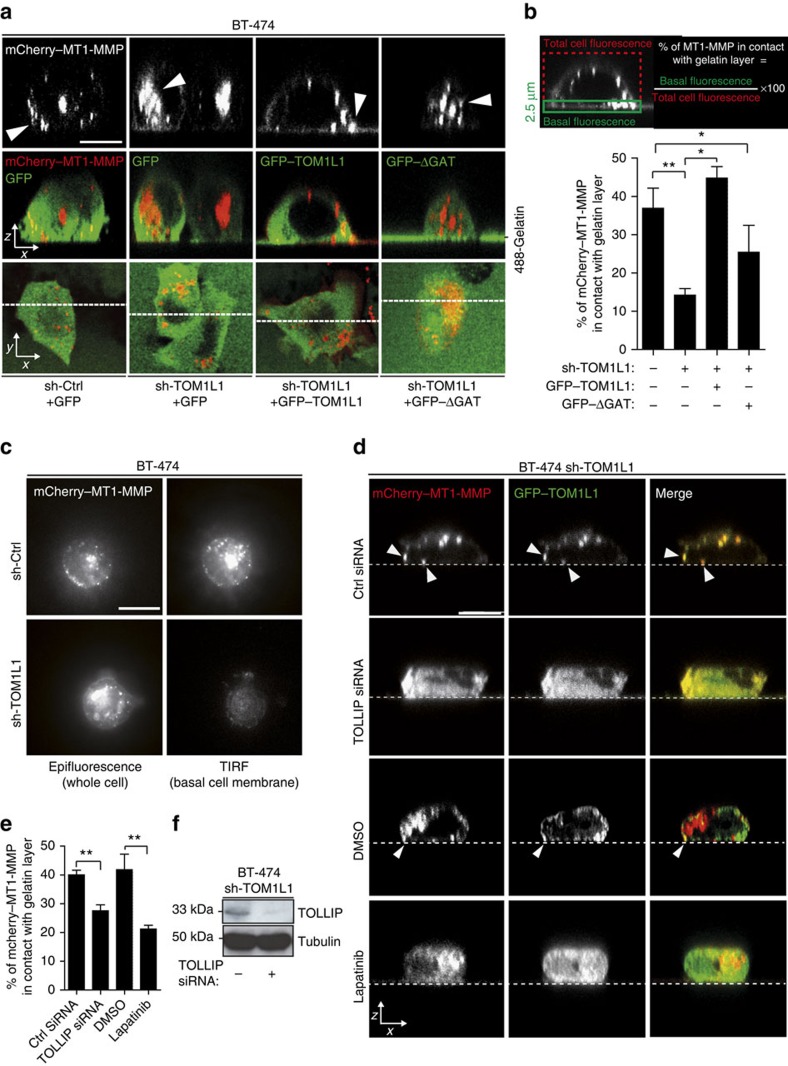
TOM1L1 regulates MT1-MMP activity. (**a**) BT-474 cells infected with shCtrl or shTOM1L1 encoding viruses were transfected with mCherry–MT1-MMP and GFP, TOM1L1–GFP or TOM1L1ΔGAT–GFP then plated on a gelatin/Oregon Green 488 gelatin mix and imaged by (*xz*) or (*xy*) confocal microscopy. Note the more basal localization of MT1-MMP when co-expressed with TOM1L1, but not with TOM1L1ΔGAT (arrowheads). Scale bar, 10 μm (See also Supplementary Movie 1). (**b**) Quantification of **a**. The fraction of mCherry–MT1-MMP in contact with the gelatin layer was evaluated as indicated in the upper panel. Lower panel shows fluorescence quantifications in indicated conditions (*n*=8). Mean±s.e.m., *P≤0.05; **P≤0.01 (Student's *t*-test). (**c**) 48 h after transfection with mCherry–MT1-MMP, BT-474 cells infected with the indicated viruses were seeded on plasma-cleaned glass coverslips coated with gelatin and imaged by epifluorescence or TIRF. Shown is a representative image out of 10. Scale bar, 10μm. (**d**) Confocal orthogonal imaging (*x/z*) of BT-474 shTOM1L1-infected cells were transfected with control (Ctrl) or *TOLLIP* siRNA, then transfected with mCherry–MT1-MMP and GFP–TOM1L1 and plated on gelatin-coated coverslips. Cells were treated with DMSO or 1 μM Lapatinib for 3 h and imaged by confocal orthogonal imaging (*x/z*). Note the strong co-localization (arrowheads) of GFP–TOM1L1 and mCherry–MT1-MMP at the basal plane (dotted lines) of cells and the loss of this localization after TOLLIP depletion or Lapatinib treatment. Scale bar, 10 μm. (**e**) Quantification of **d**. The fraction of mCherry–MT1-MMP in contact with the gelatin layer was evaluated as in **b**. Graph shows mean±s.e.m. of fluorescence quantifications in indicated conditions (*n*=4–10). **P≤0.01 (Student's *t*-test). (**f**) Western blot showing the efficiency of TOLLIP depletion 48 h after transfection of *TOLLIP* or control siRNAs in BT-474 cells.

As MT1-MMP trafficking to invadopodia plays a central role in ECM degradation^14^,^30^, we asked whether TOM1L1 mediates this cellular process. By confocal orthogonal and total internal reflection fluorescence (TIRF) microscopy, we found that TOM1L1 expression regulated MT1-MMP localization at the basal gelatin layer (Fig. 5a–c, Supplementary Movie 1). Specifically, this localization was lost in cells in which TOM1L1 was silenced (compared with cells expressing shCtrl). This defect was rescued by expression of mouse TOM1L1 (Fig. 5a,b) and required an intact TOM1L1-GAT domain (Fig. 5a,b), the presence of TOLLIP (Fig. 5d,f) and ERBB2 signalling (Fig. 5d,e). Similar results were obtained in 3T3-neu cells, where TOM1L1 induced peripheral membrane localization of MT1-MMP that was dependent on the presence of the GAT domain and on ERBB2 activity (Supplementary Fig. 7a–d). Consistent with this imaging analysis, TOM1L1 also increased the level of the catalytically active cleaved MT1-MMP isoform at the plasma membrane, while it did not affect ERBB2 membrane expression (Supplementary Fig. 7e). Finally, we found that TOM1L1 also promoted MT1-MMP exocytosis, as revealed by the increased surface accumulation of pHluorin-labelled MT1-MMP in SKBR3 and 3T3-neu cells that overexpress TOM1L1 compared with mock-transfected cells (TIRF analysis, Supplementary Fig. 8). Indeed, fluorescence of the pH-sensitive fluorescent probe pHluorin increases on exocytosis and exposure to the extracellular pH (ref. 30) (Supplementary Fig. 8 and Supplementary Movies 2 and 3). We thus conclude that TOM1L1 promotes membrane partitioning of MT1-MMP for invadopodia activity.

### TOM1L1 promotes RAB-7/MT1-MMP endosomes trafficking

**Figure 6:**
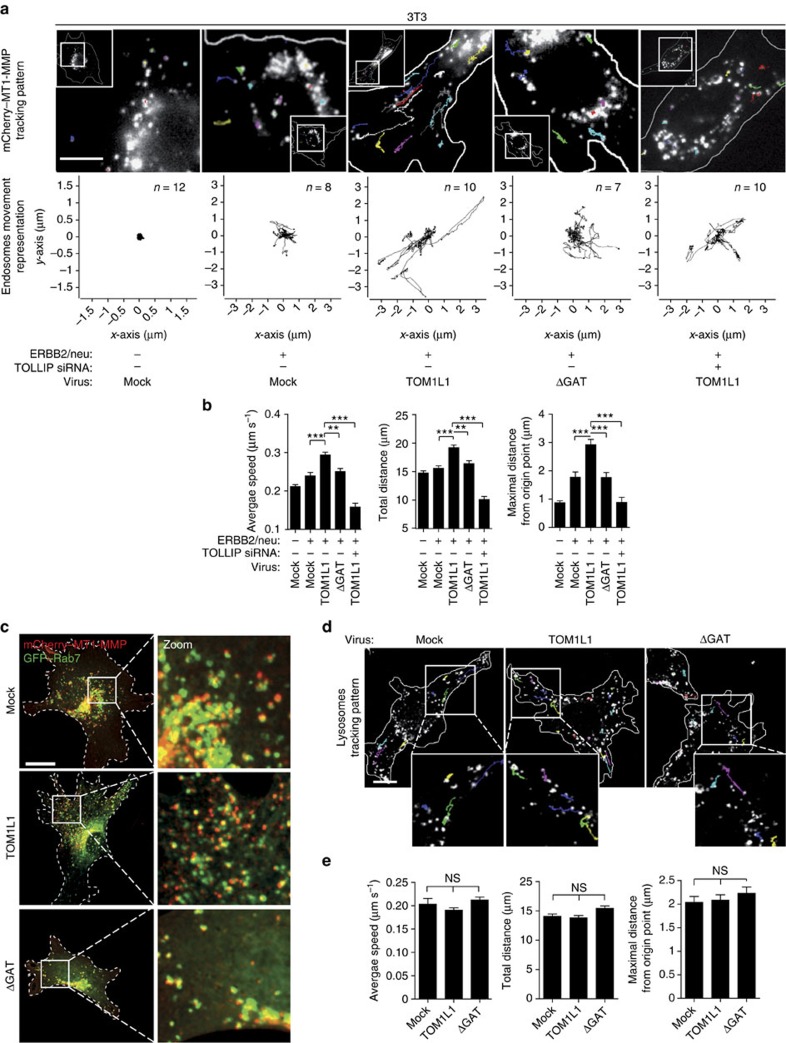
TOM1L1 specifically drives long-range trafficking of RAB-7/MT1-MMP-positive late endosomes. (**a**) Endosome tracking assay. About 48 h after mCherry–MT1-MMP transfection with Ctrl or mouse *TOLLIP* siRNA, 3T3 cells infected as indicated were plated on gelatin-coated glass bottom dishes. Time-lapse imaging was performed with one acquisition every 260 ms for 1 min to visualize endosome movements (see also Supplementary Movie 4). The track pattern of randomly selected endosomes is seen as coloured lines. Insets show the localization of the cell areas (boxed) showed at higher magnification. Scale bar, 10 μm. (**b**) Analysis of endosome tracking. Average speed (μm s^−1^), total distance (μm) and maximum distance from the point of origin (μm) were recorded. Histograms show mean±s.e.m. (*n*=101–202 tracked endosomes). **P≤0.01; ***P≤0.001 (Mann–Whitney test). (**c**) Co-localization of MT1-MMP with RAB-7. 48 h after GFP–RAB-7 and mCherry–MT1-MMP transfection, 3T3-neu cells infected with viruses expressing the indicated constructs were plated on gelatin-coated coverslips for 3 h to visualize RAB-7/MT1-MMP co-localization by confocal imaging. Boxed areas on the left panels are shown at higher magnification on the right panels. Scale bar, 20 μm (see also Supplementary Movie 5). (**d**) TOM1L1 does not affect lysosome trafficking. 3T3-neu cells infected as indicates were seeded on gelatin-coated glass bottom dishes and lysosomes were labelled with 50 nM Lysotracker-Red 30 min before imaging. Time-lapse imaging was performed with one acquisition every 260 ms for 1 min to visualize lysosome trafficking. The track pattern of randomly selected lysosomes is seen as coloured lines (see also Supplementary Movie 6). Scale bar, 20 μm. (**e**) Analysis of lysosome tracking. Average speed (μm s^−1^), total distance (μm) and maximum distance from the point of origin (μm) were recorded (*n*=160 lysosomes per condition). Histograms show mean±s.e.m/NS, no significant/(Mann–Whitney Test).

**Figure 7:**
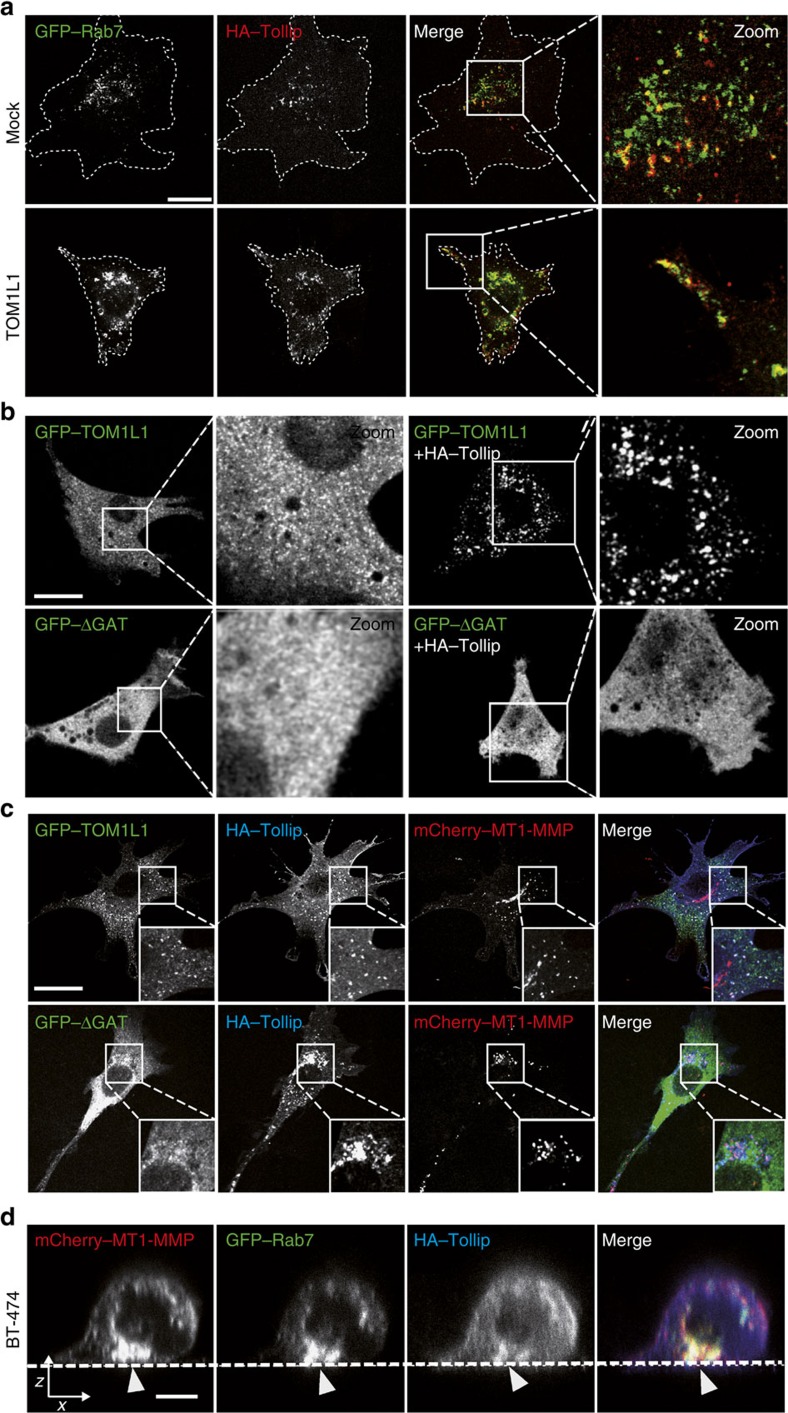
TOM1L1 is recruited by TOLLIP to RAB-7/MT1-MMP endosomes for MT1-MMP trafficking. (**a**) Co-localization of TOLLIP and RAB-7. About 48 h after GFP–RAB-7 and HA–TOLLIP transfection, 3T3-neu cells infected with viruses expressing the indicated constructs were seeded on gelatin-coated coverslips for 3 h and immunolabelled using an anti-HA antibody. ‘Zoom' panels show higher magnification of the boxed areas. Note the relocalization of TOLLIP/RAB-7 co-localization at the cell periphery when TOM1L1 is expressed. Scale bar, 20 μm. (**b**) Endosomal TOM1L1 recruitment by TOLLIP. 3T3-neu cells were transfected with GFP–TOM1L1 or GFP–ΔGAT alone or with HA–TOLLIP. About 48 h after transfection cells were seeded on gelatin-coated glass bottom dishes to visualize TOM1L1 localization. ‘Zoom‘ panels show higher magnification of the boxed areas. Scale bar, 20 μm. (**c**) Endosomal co-localization of TOM1L1, TOLLIP and MT1-MMP. About 48 h after GFP–TOM1L1/ΔGAT, HA–TOLLIP and mCherry MT1-MMP transfection, 3T3-neu cells were plated on gelatin-coated coverslips for 3 h then immunolabelled with an anti-HA antibody to visualize co-localization. Insets show higher magnification of the boxed areas. Scale bar, 20 μm. (**d**) Co-localization of MT1-MMP, RAB-7 and TOLLIP in BT-474 cells. BT-474 cells were transfected with mCherry–MT1-MMP, GFP–Rab-7 and HA–TOLLIP. About 48 h after transfection, cells were plated on gelatin-coated coverslips and imaged by confocal orthogonal (*x/z*) imaging. Note the basal co-localization of mCherry–MT1-MMP, GFP–RAB-7 and HA–TOLLIP (arrowheads). Scale bar, 10 μm.

We next started investigating the underlying mechanism of MT1-MMP trafficking in ERBB2-transformed cells. Overexpression of TOM1L1 in 3T3-neu cells led to increased long-range bi-directional trafficking of mCherry–MT1-MMP, based on quantification of the average speed, total distance travelled by the vesicles and maximal distance from the point of origin (that is, directionality) (Fig. 6a,b and Supplementary Movie 4). This cellular process involved microtubules, as shown in Supplementary Movie 5, with the use of the microtubule-targeting drug Paclitaxel that inhibits TOM1L1-induced MT1-MMP and RAB-7-containing vesicles trafficking. Indeed, MT1-MMP strongly co-localized with RAB-7 and moved through RAB-7-decorated late endosomes/lysosomes (LE/Ly) (Fig. 6c and Supplementary Movies 5 and 6, left panel). Interestingly, TOM1L1 specifically induced peripheral scattering of RAB-7- and MT1-MMP-containing vesicles, but did not affect the distribution of RAB11- or RAB5-decorated vesicles (Supplementary Fig. 9) or the global LE/Ly trafficking (Fig. 6d,e and Supplementary Movie 6). These data are consistent with a TOM1L1 specific role in the long-range trafficking of RAB-7/MT1-MMP-positive late endosomal compartments. This cellular process also involved TOLLIP because this sorting protein co-localized with RAB-7-containing vesicles (Fig. 7a), recruited TOM1L1 to endosomes in a GAT-dependent manner (Fig. 7b) and strongly co-localized with co-expressed MT1-MMP and TOM1L1, but not with TOM1L1 ΔGAT (Fig. 7c). In addition, TOM1L1 promoted the peripheral distribution of TOLLIP/RAB-7-containing vesicles also in BT-474 cells (Fig. 7d). Altogether, these data support a model where TOLLIP docks TOM1L1 at RAB-7/MT1-MMP-positive endosomes to favour targeting of these endosomes at ECM proteolytic degradation sites.

### TOM1L1 phospho-Ser321 regulates interaction with TOLLIP

**Figure 8:**
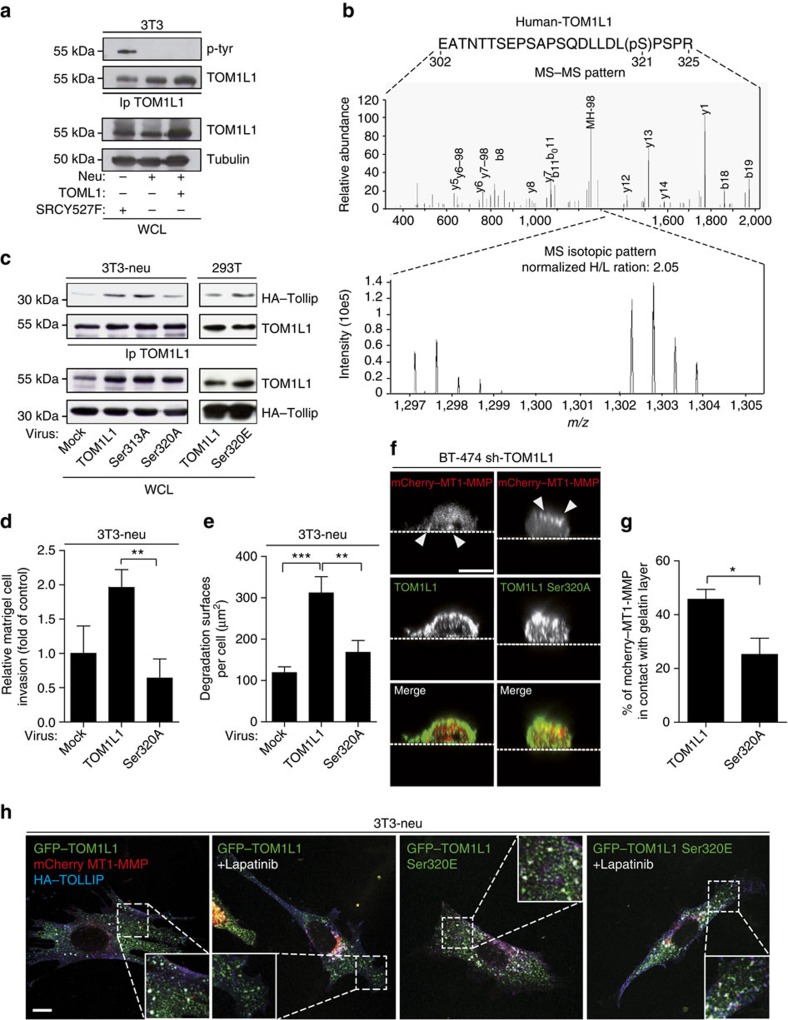
ERBB2 indirectly promotes TOM1L1-Ser321 phosphorylation for interaction with TOLLIP and MT1-MMP trafficking. (**a**) Lysates from 3T3 cells infected as indicated were immunoprecipitated with an anti-TOM1L1 antibody and immunoblotted to visualize TOM1L1 phosphorylation. (**b**) SILAC mass spectrometry analysis. 3T3-neu cells transfected with GFP–hTOM1L1 were cultured for 2 weeks in medium containing light (^12^C_6_^14^N_4_-Arg and ^12^C_6_^14^N_2_-Lys) or heavy (^13^C_6_^15^N_4_-Arg and ^13^C_6_^15^N_2_-Lys) arginines and lysines and treated with 1 μM Lapatinib or not (DMSO) for 3 h before lysis. GFP–hTOM1L1 was immunoprecipitated using the GFP-Nanotrap technology then digested using trypsin. The hTOM1L1 peptides phosphorylation was then analysed by mass spectrometry (see Methods for details). Upper panel: fragmentation spectra of the single peptide find phosphorylated (localization on Ser321 with a probability >0.75 as calculated by MaxQuant). Lower panel: Heavy/Light SILAC ratio (H/L) for this peptide, traducing the phosphorylation ratio changes between the tested conditions. (**c**) 3T3-neu and 293 T cells infected as indicated were transfected with HA–TOLLIP. Lysates were then immunoprecipitated and immunoblotted as shown. (**d**) 3T3-neu cells infected as indicated were seeded in Boyden chambers with matrigel for 24 h and cells present in the lower chamber were counted. The histogram shows the invasion ratio normalized to control (*n*=3). **P≤0.01 (Student's *t*-test). (**e**) 3T3-neu cells infected as indicated were cultured on Oregon Green 488 gelatin for 24 h to visualize gelatin degradation areas. The quantification (mean±s.e.m.) of degradation areas per cell is shown (*n*=26-48). **P≤0.01; ***P≤0.001 (Student's *t*-test). (**f**) BT-474 cells infected with TOM1L1 shRNA and transfected with indicated constructs were imaged by confocal orthogonal imaging. Arrowheads show the change of MT1-MMP apico-basal polarity. Scale bar, 10 μm. (**g**) Quantification of **f**. The fraction of mCherry–MT1-MMP in contact with the gelatin layer was evaluated as in Fig. 5b (*n*=4). Mean±s.e.m. *P≤0.05 (Student's *t*-test). (**h**) 3T3-neu cells transfected with mCherry–MT1-MMP, HA–TOLLIP and GFP–TOM1L1 or the phosphomimetic mutant (S320E) were treated or not with 1 μM Lapatinib for 2 h. Localization of colocated spots was visualized by confocal imaging. Insets show higher magnification of the boxed areas. Scale bar, 10 μm.

**Figure 9:**
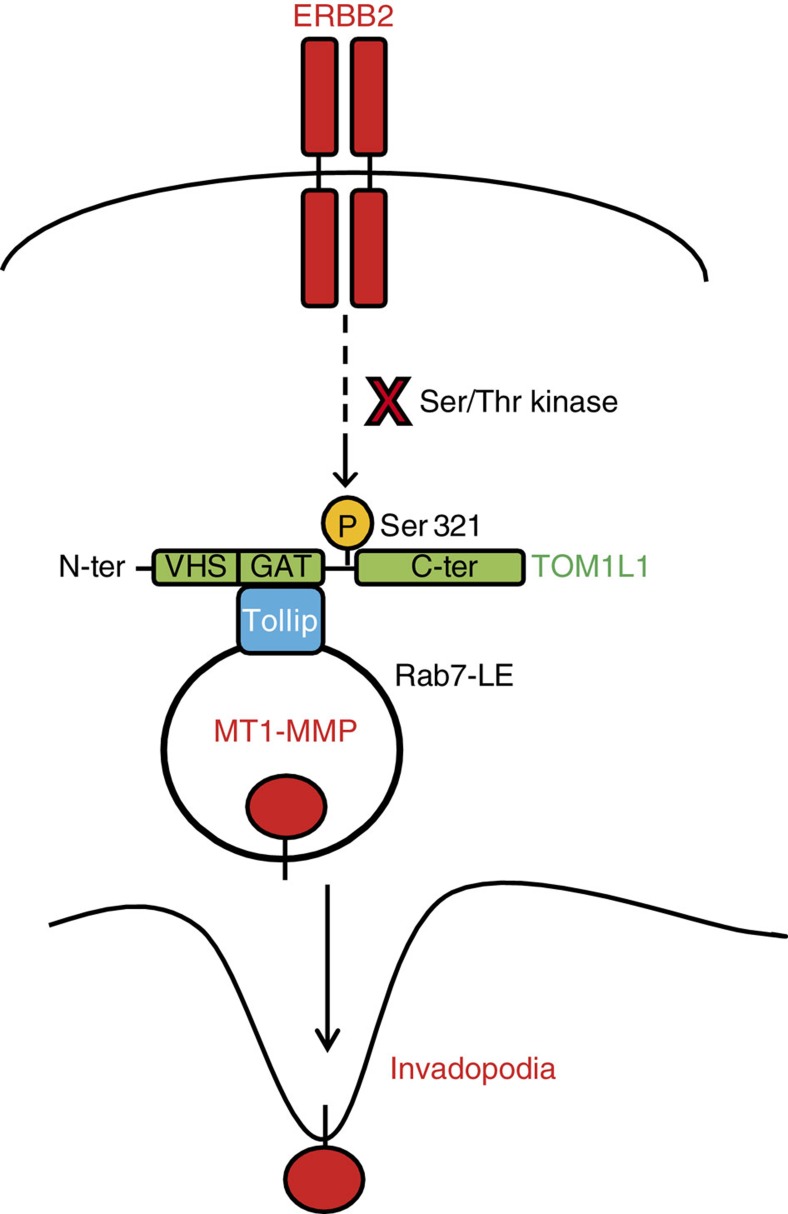
A model for TOM1L1 invasive activity. ERBB2 indirectly induces TOM1L1 phosphorylation at Ser321 to promote association with TOLLIP in RAB-7/MT1-MMP-positive late endosomes and MT1-MMP trafficking to plasma membrane for cell invasion.

Our results indicate that TOM1L1 overexpression alone is not sufficient to induce invasion, but requires an ERBB2-dependent signal. We thus studied how ERBB2 signals to TOM1L1. In contrast to SRC, ERBB2 did not induce tyrosine phosphorylation of TOM1L1 (Figs 3c and 8a), suggesting that ERBB2-induced TOM1L1 activation is mediated by another, yet unidentified mechanism. Available phospho-proteomic data (www.phosphosite.org) revealed that TOM1L1 can also be phosphorylated on serine residues, including Ser314 and Ser321 (Ser313 and Ser320 in the mouse sequence). These serine residues are in the TOM1L1 linker region that may behave as a hinge to control TOM1L1 conformation and GAT access to its partners, such as TOLLIP, as described for the related adaptor protein GGA3 (ref. 31). Quantitative phospho-proteomic analysis of ERBB2-induced TOM1L1 phosphorylation in fibroblasts did not detect any tyrosine phosphorylation, but revealed phosphorylation at Ser321 and Ser323. In addition, only Ser321 phosphorylation level was reduced (≈50%) on ERBB2 inhibition by Lapatinib (Fig. 8b and Supplementary Table 4). We thus conclude that ERBB2 induces TOM1L1 phosphorylation at Ser321 via an unidentified Ser/Thr kinase. We next addressed the biological relevance of this phosphorylation on ERBB2-induced TOM1L1 activity. Unlike Ser313 (not found phosphorylated in proteomics and used as control), mutation to Alanine of Ser320 (S320A), the residue corresponding to Ser321 in mouse TOM1L1, reduced TOLLIP binding (Fig. 8c). Furthermore, overexpression of the TOM1L1 S320A mutant reduced cell invasion (Fig. 8d) and ECM degradation in 3T3-neu cells compared with wild-type TOM1L1 (Fig. 8e). Similarly, it could not rescue TOM1L1-silencing defect (in this case localization of MT1-MMP at the basal plasma membrane) (Fig. 8f,g). Conversely, phosphomimetic mutation of Ser320 into Glutamic acid (S320E) increased TOM1L1 interaction with TOLLIP in the ERBB2-negative 293T cell line (Fig. 8c). This phosphomimetic mutant also strongly promoted the peripheral localization and co-localization of TOM1L1 with TOLLIP and MT1-MMP in 3T3-neu cells compared with wild-type TOM1L1 (Fig. 8h). Differently, from wild-type TOM1L1, the localization effect of the S320E TOM1L1 mutant was not affected by ERBB2 kinase inhibition with Lapatinib (Fig. 8h), suggesting that phosphorylation of Ser321 defines the main mechanism by which ERBB2 signals to TOM1L1. Overall, our data are consistent with an indirect ERBB2-induced phosphorylation of TOM1L1 at Ser321 that triggers its GAT-dependent association with TOLLIP to favour MT1-MMP membrane delivery for ECM degradation and invasion (proposed model in Fig. 9).

## Discussion

Vesicular trafficking proteins play central roles in the control of cell surface receptor signalling via receptor endocytosis and sorting for lysosomal degradation in normal cells. Consequently, deregulation of the endocytic machinery can promote abnormal receptor signalling that leads to malignant cell transformation^1^. In support to this model, several components of this biological process display tumour suppressor function, as demonstrated by the original identification of the endosomal protein TSG101 in human breast cancer. Conversely, our study demonstrates a pro-tumoural function for the GAT-containing trafficking protein TOM1L1 in human breast cancer and shows that its pro-oncogenic activity is induced by gene co-amplification with the *ERRB2* oncogene. In addition, we have identified one mechanism involved in this process that involves the efficient translocation of MT1-MMP from endosomes to the plasma membrane in invadopodia structures for the promotion of cancer cell invasion. The findings that this TOM1L1 pro-invasive activity is present also in transformed fibroblasts and that *TOM1L1* is amplified in other cancer types (www.bioportal.com) suggest that TOM1L1 upregulation may define a more general mechanism of metastatic progression. Interestingly, the GAT-containing protein GGA3 has been recently involved in efficient MET receptor recycling from RAB4-positive endosomes, for the promotion of sustained ERK activity and cell migration^32^. Moreover, MET endocytic signalling plays a central role in tumorigenesis^33^. Whether GGA3 plays a role in this malignant process has not been addressed yet, but available genomic data highlighted *GGA3* amplification in some human tumours (www.cbioportal.org). We thus anticipate additional pro-tumoural functions of GAT-containing proteins in human cancer. As ERBB2 trafficking is impaired in tumour cells^34^, it is unlikely that a GGA3-dependent mechanism may apply to TOM1L1. Accordingly, we found a very specific effect of TOM1L1 on cell invasion. This effect seems inconsistent with a global alteration in ERBB2 cell surface abundance and signalling as it would also inhibit ERBB2-induced cell proliferation and migration. Moreover, we did not detect any modification in ERBB2 membrane localization induced by TOM1L1 (Supplementary Fig. 7e). Thus, GAT-containing proteins may participate in cell transformation through several mechanisms.

Our results are also inconsistent with the established negative role of TOM1L1 in SRC-mitogenic signalling and raise the question about the mechanism that governs these opposite functions. One hypothesis involves phosphorylation of TOM1L1 on specific residues. TOM1L1 inhibitory functions could be linked to tyrosine phosphorylation and association with CHC, while its pro-tumoural activity could be dependent on serine phosphorylation and association with TOLLIP. It would be interesting to identify the serine kinase(s) involved in this process. CDC42-binding protein kinase beta, p21-activated protein kinase 4 (ref. 12) and HUNK^9^ are downstream effectors of ERBB2 that could be interesting candidates. Recently, the atypical protein kinase Cι (PKCι) has been identified as a key regulator of MT1-MMP trafficking in breast cancer^35^ and could also be an interesting candidate. It is not known why TOM1L1 is not tyrosine-phosphorylated in ERBB2-positive cancer cells. One hypothesis is that ERBB2 might not require SRC-like activity for invasive signalling. Actually, the role of SRC family kinases in ERBB2 signalling is controversial^36^ and constitutive ERBB2 kinase activity may overcome, at least in part, the need of SRC signalling by direct phosphorylation of its downstream substrates. For example, cortactin may be a direct ERBB2 substrate for the promotion of invasive structures, such as invadopodia^37^.

Our report also shows that ERBB2 expression promotes invadopodia formation in breast cancer cells. This is in accordance with data demonstrating that proteolysis is required for ERBB2 invasive signalling^10^,^11^,^12^. Specifically, the extracellular activity of cysteine cathepsins is required for ERBB2-driven invasion^12^. As these enzymes are localized in lysosomes, exocytosis might be involved in the process connected with invadopodia formation^38^. TOM1L1 favours ERBB2 invasive signalling by amplifying MT1-MMP trafficking to the plasma membrane via interaction with TOLLIP in a RAB-7-positive LE compartment. This is in accordance with previous data showing co-localization of MT1-MMP with VAMP7, a transmembrane protein that localizes to LE/Ly^39^, and with two recent report showing MT1-MMP trafficking from RAB-7-positive LE/Ly to invadopodia^39^,^40^. As already described for TOM1 (ref. 41), we found that TOLLIP regulates TOM1L1 docking to RAB-7- and MT1-MMP-positive endosomes and then promotes MT1-MMP trafficking to invadopodia. In this model, TOM1L1 could function in the same way as its homologue TOM1 (ref. 42). Indeed, TOM1 associates with the actin-based molecular motor Myosin IV, which then delivers TOM1-positive endosomal membranes to autophagosomes by docking to optineurin, NDP52 or T6BP, thus facilitating autophagosome maturation. Here we have not identified the molecular motor involved in TOM1L1-induced MT1-MMP trafficking, but proteins from the kinesin family could be involved in this process. Indeed, ERBB2 regulates microtubule dynamics^6^,^7^ and it was recently shown that MT1-MMP transport along microtubules is regulated by the kinesins KIF5B and KIF3A/KIF3B in leucocytes^43^. TOM1L1 and TOLLIP are both ubiquitin-binding proteins and TOM1L1 is a member of the alternate ESCRT 0 complex that sorts ubiquitinated proteins from LE to Ly^19^. As MT1-MMP can be mono-ubiquitinated^44^, this post-translational process might also play a role in this TOM1L1 trafficking function.

Our results show that *ERBB2* and *TOM1L1* are frequently co-amplified and define a subgroup of patients with ER-positive cancer with worse prognosis. Crosstalk between ER, ERBB2 and invasive signalling is well-known. For example, oestrogen signalling inhibits the invasive phenotype by repressing RELB and its target *BCL2* (ref. 45). Conversely, cell transformation by ERBB2 can affect ER signalling as it induces tamoxifen resistance^46^. Co-amplification of *TOM1L1* and *ERBB2* significantly reduces survival of patients with ER-positive cancers. This suggests a potential function of TOM1L1 in a positive selection process, possibly through induction of epithelial-to-mesenchymal transition that is inhibited by ER. Thus, it would be interesting to test whether TOM1L1 might affect the ERBB2-ER crosstalk and the resulting signalling balance.

In conclusion, we show that ERBB2 exploits the trafficking function of TOM1L1 to promote cancer cell invasion. These results highlight that regulators of protein trafficking are important elements of oncogenic signalling induced by tyrosine kinases. Moreover, the amplification of the corresponding genes represents a novel mechanism to promote metastatic progression. The targeting of this signalling pathway may be of therapeutic value in this kind of cancers.

## Methods

### Primary breast tumour samples

A set of 402 primary breast tumour DNA samples and 84 RNA samples purified from a subset of the 402 tumours were obtained from the Pathology Department of the Montpellier Cancer Hospital (ICM) (France). This study has been approved by the institutional review board CORT (Comité de Recherche Translationelle) of the ICM hospital and informed consent was obtained from the patients. This series of 402 breast tumours included invasive ductal carcinomas (67.7%), invasive lobular carcinomas (19.7%), invasive adenocarcinomas (7%) and other histological types (5.5%). Grading according to the Scarff-Bloom and Richardson classification was as follows: 10.1% grade 1, 53.8% grade 2 and 36.1% grade 3. The patients' mean age was 57.5 years. Radioligand-binding assay indicated that 69.7% of cancers were ER+ (≥10 fmol mg^−1^ of protein) and 71.9% progesterone receptor positive (PR+).

### Array-CGH

Array-CGH data corresponded to the data set described in ref. 18. Gpr files were loaded in the Nexus 6.0 software (Biodiscovery, El Segundo, CA, USA) to perform array-CGH profile analysis. Analysis settings for data segmentation and calling were as follows: significant threshold for the Rank Segmentation algorithm: 0.005, Max Continuous Probe spacing: 6,000, Min Number of probes per segment: 6, gain: 0.25, loss: −0.25. Nexus 6.0 was used to calculate and draw individual profiles, frequency plots, Kaplan–Meier plots and log-rank tests.

### Real-time quantitative PCR

Reverse transcription was performed using 1 μg of total RNA that had been pre-treated with RNase-free DNase (Promega, France), the SuperScript II RT and 250 ng of random hexamers (Invitrogen, France). Quantitative PCR reactions were carried out with an ABI Prism 7000 instrument (Applied Biosystems, France) in a final volume of 15 μl following the manufacturer's conditions using SYBR Green as a detector. Primers (see Supplementary Table 5 for sequences) were designed with the assistance of the Primer Express software (Applied Biosystems). Each gene was quantified at least twice. Standard curves were determined for each gene by using serial dilutions of the same pool of complementary DNA (cDNA) and/or genomic DNA. Relative quantities were calculated by using these curves. The relative expression level of each target gene was normalized to the 28S endogenous reference. RNA variations were then determined by calculating the ratio of the normalized value of each gene to the normalized values obtained from six normal breast RNA samples. Ratios exceeding 1.8 in tumour samples were considered as overexpression and ratios <0.55 in tumours as underexpression. Similarly, the relative genomic level of each target gene was normalized to the median of the reference genes *ALB/DCK/GAPDH* and copy number variations were determined by calculating the ratio between the normalized value of each sample and the median value of all tumours. The same thresholds as before were applied after checking *ALB*, *DCK* and *GAPDH* variations.

### Immunohistochemistry

IHC analyses were performed using 3-μm-thin tissue microarray (TMA) sections including 108 breast tumours encompassing the 4 major subtypes (ER+HER2+, ER+HER2−, ER−HER2+ and ER−HER2−). Formalin-fixed paraffin-embedded breast tumours were sampled in triplicate tissue cores (0.6 mm in diameter), taken from three different malignant areas, using a manual arraying instrument (MTA, Beecher Instrument, USA) and as described^47^. Following antigen retrieval with the K8004 buffer (Dako, Denmark), TMA sections were incubated with monoclonal antibodies against TOM1L1 on an Autostainer Link48 platform (Dako) using the Flex+ system for signal amplification and DAB (diaminobenzidine tetrahydrochloride) as chromogen. Slides were covered and observed under a light microscope. Each spot in the TMA sections was evaluated for staining intensity (categorized as 0 (absent), 1 (weak), 2 (moderate), or 3 (strong), see Fig. 1) and for the percentage of marked cells (ranging from 0 to 100%). Data were then consolidated as the mean of the triplicate values. Finally, a Quick Score (QS), ranging from 0 to 300, was defined by multiplying the intensity grade by the percentage of stained nuclei. This overall score for each tumour was further simplified by dichotomizing it to negative (QS<20) or positive (QS≥20).

### Antibodies

Polyclonal anti-TOM1L1 (1: 2,000) antibodies were as described in ref. 23. The monoclonal anti-TOM1L1 antibody was from Covalab (Lyon, France). Antibodies against ERBB2 (1:1,000), p-CortactinY421 (1:500), p-AKTS473 (1:1,000), AKT (1:1,000), P44/P42 MAPK (1:1,000) and p-P44/P42 MAPK (1:1,000) were from Cell Signaling Technology (Danvers, USA). Antibody against Cortactin (1:500) was from Millipore (Billeria, USA) and antibodies against MT1-MMP (1:100 for if or 1:500 for immunoblotting) and TOLLIP (1:200) from Abcam (Cambridge, UK). Anti-tubulin (1:2,000), HA-tag (1:4,000) and p-Tyr 4G10 (1:50) antibodies were from N. Morin, C. Gauthier-Rouvière and P. Mangeat, respectively (CRBM, Montpellier, France). Anti-rabbit IgG-HRP (1:5,000) and anti-mouse IgG-HRP (1:5,000) were from GE Healthcare (Fairfield, USA). Anti-rabbit and anti-mouse IgG coupled to Alexa-Fluor 488, Alexa-Fluor 594 and Alexa-Fluor 405 (1:1,000) were from Life Technologies (Carlsbad, USA). Alexa-Fluor 594-Phalloidin (Life Technologies) was used to visualize F-actin. GFP-Nanotrap technology antibodies were from Chromotek (Planegg-Martinsried, Germany).

### Cell culture and inhibitors

All cell lines were obtained from the American Type Culture Collection (ATCC, Rockville, MD, USA) and cultured in Dulbecco's modified Eagle's medium (DMEM) or RPMI 1,640 medium supplemented with 10% foetal calf serum (FCS), glutamine and antibiotics (gentamicin, penicillin and streptomycin) at 37 °C in a humidified 5% CO_2_ atmosphere. Stable cell lines were obtained by selection with 0.5–1 μg ml^−1^ puromycin or 200 μg ml^−1^ hygromycin B. Cell culture reagents were from Life Technologies. For inhibitor assays, cells were treated with 5 μM SU6656 (Merck-Millipore), 1 μM Lapatinib (Merck-Millipore), 1 μM AG879 (Sigma-Aldrich, Saint-Louis, USA), 12.5 μM GM6001 (Merck-Millipore) and 2 μM Paclitaxel (Sigma-Aldrich) for 2–3 h.

### Transfections and retroviral infections

Transient transfections were performed with the jetPEI reagent (Polypus Tranfection, Illkirch, France) (cDNA vectors) and with Lipofectamine Plus reagent (Invitrogen, Carlsbad, USA) (siRNAs), according to the manufacturer's instructions. Cells were transfected 48 h before imaging or lysis. Retroviral infection procedures were as described in ref. 25.

### DNA constructs and mutagenesis

pBABE constructs encoding murine TOM1L1, TOM1L1-ΔLinker (deletion of amino acids 292–386) TOM1L1-ΔLinker/L401A, TOM1L1-ΔLinker/YFPP (R419D/P421A/P424A/Y457F), TOM1L1-ΔC-ter (deletion of amino acids 388–474), TOM1L1-ΔGAT (deletion of amino acids 157–284), TOM1L1-ΔVHS (deletion of amino acids 1–153), TOM1L1 Ser313A, TOM1L1 Ser320A and TOM1L1 Ser320E were obtained by PCR using the QuikChange Site-Directed Mutagenesis System (Stratagene, La Jolla, USA). Mouse and human green fluorescent protein (GFP)–TOM1L1, GFP–ΔGAT, GFP–Ser320A and GFP–Ser320E were obtained by subcloning TOM1L1 constructs in pEGFP. Constructs encoding HA–TOLLIP (pcDNA3), mCherry–MT1-MMP (pcDNA3) and MT1-MMP pHluorin, Luciferase (pcDNA3.1) were from E. Lemichez, P. Chavrier and P. Balaguer, respectively. GFP–RAB-7, GFP-Rab-5 and GFP–RAB11 (pEGFP) were from C. Gauthier-Rouvière.

### shRNA and siRNA duplexes

Mock or short hairpin RNA (shRNA) specific to human *TOM1L1* were cloned in the pSiren retroviral vector (mock: 5′-GACACTCGGTAGTCTATAC-3′; sh-*TOM1L1-1*: 5′-ACAAGAGACTGCTCAAAT-3′ or sh-*TOM1L1-2*: 5′-CAGAAGGAAGCCAATA-3′). The siRNA specific for human *TOLLIP* (sequence: 5′-AAGTTGGCCAAGAATTACGGCdTdT-3′) was designed with the Qiagen design tool and obtained from Qiagen (Venlo, Netherlands). Control and siRNA specific for mouse *TOLLIP,* mouse and human *MT1-MMP* were from GE Healthcare (Dharmacon), Fairfield, USA.

### Migration and invasion assays

Cell migration and invasion assays were performed as described in ref. 48. Briefly, (20,000 cells were used for migration assays) and 50,000–80,000 for invasion assays. Cells were fixed after 45 min (migration) or 24–48 h (invasion) and counted.

### Spheroid invasion assay

Spheroid invasion assay was done essentially as described in ref. 49. In brief, 1,000 cells were plated in medium containing 2.4 mg ml^−1^ methylcellulose in a well of a 96-well plate with round bottom and incubated for 24 h to make spheroids. Then, spheroids were embedded in a mixture (2:1) of neutralized bovine collagen I (2 mg ml^−1^) and matrigel (3 mg ml^−1^) or in neutralized rat tail intact telopeptides collagen I (1.7 mg ml^−1^) and placed in a well of a 96-well plate flat bottom covered with 50 μl of neutralized bovine collagen I or rat tail collagen 1, respectively. Invasion was followed by time-lapse microscopy using an inverted fluorescent microscope Leica DMIRE2 equipped with a Leica × 10 C PLAN 0.22 LMC in DMEM/10% FCS under CO_2_ and temperature controls every hour for 24–48 h (3T3 cells), 72–75 h (SKBR3) and 75 h–5 days (BT-474). Invasive fronts were defined as three to six layers of cells progressing into the matrix.

### Invadopodia gelatin degradation assay

Invadopodia degradation assays were performed as described^48^,^50^. Briefly, coverslips were incubated with 50 μg ml^−1^ poly-D-lysine, then with 0.5% glutaraldehyde and then inverted on a 20 μl drop of gelatin+Oregon Green 488–conjugated gelatin (Life technologies) (10:1) mixture. Gelatin matrix were then quenched with 5 mg ml^−1^ sodium borohydride and rehydrated in complete growth medium before use.

### Wide-field and confocal imaging

Wide-field imaging was used to follow invadopodia gelatin degradation assays using Zeiss AxioimagerZ1 or Zeiss AxioimagerZ2 upright microscopes with Zeiss × 10 Plan Apo 0.45, Zeiss × 10 EC Plan Neofluar 0.3, Zeiss × 40 EC Plan NeofluaR 1.3 oil DIC or Zeiss × 63 Plan-Apochromat 1.4 oil objectives. Image acquisition and quantification of degradation surface areas were carried out using Metamorph. For confocal imaging, cells were cultured on gelatin-coated coverslips for 3 h and imaged using confocal Leica SP5-SMD or confocal Zeiss LSM780 multi-photon microscopes and × 63/1.4 Oil DIC Plan-Apo or Leica × 63/1.4 Oil HCX PL APO CS objectives. Images were acquired using the LAS-AF (Leica, Wetzlar, Germany) or Zeiss Zen 2010 (Zeiss, Oberkochen, Germany) softwares. Brightness, contrast and median filter (0.5 to 1 pixel radius) adjustments of images were realized using the imageJ software. Fluorescence quantifications in Figs 5b,e and 8g were done using imageJ software and the corrected cell fluorescence formula (CCF=integrated density−(area of selected cell × mean fluorescence of background readings)^51^. For live confocal imaging, cells were cultured on gelatin-coated glass bottom dishes (Ibidi, Plannegg Martinsried, Germany) for 2 h. Cells were imaged in a humidified atmosphere of 5% CO_2_ at 37 °C using a confocal Leica SP5-SMD microscope with Leica × 63/1.4 Oil HCX PL APO CS or Leica × 40/1.3 Oil HCX PL APO CS objectives. Rapid acquisition was done by using a high-speed resonant scanner (8,000 Hz) (Leica) to avoid photo-bleaching and toxicity. Imaging of pHluorin-tagged MT1-MMP (Supplementary Movies 2 and 3) was done using a homemade TIRF set-up based on a Zeiss Axiovert 200 inverted microscope, equipped with an alpha Plan-Fluar × 100/1.45 NA objective (Zeiss) and with a 488 nm Argon ion blue laser (Spectra physics, Santa Clara, USA). Fluorescence signals were collected through the same objective, passed through a filter cube containing a dichroic mirror transmitting the 510 wavelengths (Chroma, Vermont, USA) and imaged onto an EM-CCD camera (Andor iXon, Belfast, Ireland). The laser power was controlled by an acoustic–optic tunable filter. The camera exposure time used was 100 ms. Movies were reconstructed using ImageJ.

### Endosomes and lysosomes tracking assay

Cells expressing mCherry–MT1-MMP or labelled with 50 nM LysoTracker-Red (Life Technologies) were plated on gelatin-coated glass-bottom dishes and imaged using a Nikon TE Eclipse microscope with a Nikon × 100 PL APO VC 1.4 oil objective or a confocal Leica SP5-SMD with a Leica × 63/1.4 Oil HCX PL APO CS objectives. Images were taken every 230 ms for 1 min. mCherry–MT1-MMP endosomes or LysoTracker-labelled lysosomes were tracked using 15 × 15 pixel and 25 × 25 pixel research boxes (Metamorph ‘Track object' function). Endosomes 114 (3T3-neu mock), 202 (3T3-neu TOM1L1), 101 (3T3-neuΔGAT), 108 (3T3-neu TOM1L1 ctrl-siRNA) and 112 (3T3-neu TOM1L1 *TOLLIP* siRNA) were tracked in a minimum of 10 different cells. For lysosome tracking, ≈160 different lysosomes in a minimum of 5 different cells per condition were tracked. Speed (μm s^−1^), distance (μm) and maximal distances from the origin point (that is, directionality) (μm) were recorded. The endosome movement representations were done using the Chemotaxis and Migration Tool 2.0 software (Ibidi).

### Standard proliferation assay

Cell proliferation was measured *in vitro* using the Sulforhodamine B assay for cytotoxicity screening (Sigma-Aldrich). Briefly, 50,000 cells per well were seeded in 24-well plates with 2% FCS medium and then fixed every day (during 4 days) in 2 × 6.1 N TCA solution at 4 °C for 1 h. Cell proliferation was evaluated by protein labelling with 0.4% Sulforhodamine B solution and optical density reading at 490 nm.

### Biochemical assays

Immunoprecipitation experiments and western blotting were performed as described in ref. 5. Briefly, cells were rinsed twice in PBS and scraped in 2 × lysis buffer containing 1% Triton X-100, 10 mM Tris-HCl (pH 7.5), 150 mM NaCl, 5 mM EDTA, 75 U ml^−1^ aprotinin and 1 mM vanadate. Immunoprecipitations were done using 800 μg–1 mg protein extracts and specific antibodies; 20–50 μg proteins were used as whole cell lysate (WCL). Biotinylation of cell surface ERBB2 and MT1-MMP was done as described in ref. 52. Immunoprecipitates and WCL were separated on 7.5/9/10% SDS–PAGE gels and transferred onto Immobilon membranes (Millipore Molsheim, France). Detection was performed using the ECL System (GE Healthcare). Larger images of all immunoblots shown in the main article are included in Supplementary Fig. 10.

### Experimental brain and bone metastasis assay

All procedures were performed in accordance with the National Committee of Ethical Thinking on Animal Experimentation, according to protocols approved by the Languedoc Roussillon Animal Care and Use Committee (registration number: CEEA-LR-12072) in an accredited establishment (Agreement No. C34-172-27). Intracardiac injection and bioluminescence detection were performed as in ref. 53. Briefly, 0.5 × 10^6^ neu-NIH-3T3 cells or 1 × 10^5^ HCC-1954 cells in 50 μl of sterile Dulbecco PBS without Ca2+ and Mg2+ were injected in the heart left ventricle of 6-week-old female BALB/c nude or athymic mice, respectively (Harlan Laboratories, Le Malourlet, France). Mice were anesthetized with 2% isofluorane/air mixture. A total of 12 animals per cell line (3T3-neu mock, 3T3-neu TOM1L1 and 3T3-neu ΔGAT cells; HCC-1954 mock, HCC-1954 TOM1L1 and HCC-1954 ΔGAT cells) were used in two independent experiments. For bioluminescence detection, mice were anesthetized with a 2% isofluorane/air mixture and a single dose of 150 mg kg^−1^ D-luciferin (Promega, Madison, USA) in PBS was administered intraperitoneally. For photon flux counting, a charge-coupled device camera system with a nose-cone isofluorane delivery system and heated stage for maintaining body temperature were used. Imaging was completed between 5 and 10 min after luciferin injection. Results were analysed after 1–5 min of exposure using a Xenogen IVIS Lumina calliper CCD camera coupled to the Living Image Acquisition and Analysis software (Caliper, Kopkinton, USA). Signal intensity was quantified for the whole animal and for distal metastases in head and legs detected by photon flux. For *ex vivo* imaging, mice were killed immediately after luciferin injection and *in vivo* imaging and then dissected to image brain, legs, heart (as a cell injection control), lungs and ribs (not shown).

### SILAC and phospho-proteomic analysis

SILAC (^13^C_6_^15^N_4_-Arg and ^13^C_6_^15^N_2_-Lys as heavy amino acids) and tryptic digestion were performed essentially as previously described in ref. 54. Cells were transfected with 7 μg of the GFP–hTOM1L1 construct 2 days before lysis and incubated with 1 μM Lapatinib 3 h before lysis (the first time in light culture conditions and the second time in heavy culture conditions for biological replicates). GFP–hTOM1L1 was purified using the GFP-Nanotrap technology for efficient immunoprecipitation specificity and to avoid the presence of immunoglobulin heavy chains in samples. Immunoprecipitates were separated on SDS–PAGE gels, and trypsin-digested samples obtained from GFP–hTOM1L1 cut gel slices were analysed essentially as described in ref. 53. Briefly, samples were analysed using nanoHPLC (Ultimate 3000, Dionex)/nanoelectrospray ionization on an orbitrap mass spectrometer (LTQ-Orbitrap XL, Thermo Fischer scientific). Sample desalting and pre-concentration were carried out online using a Pepmapper column (0.3 × 10 mm, Dionex). A gradient consisting of 0−40% *B* for 60 min, 40−80% *B* for 15 min (*A*=0.1% formic acid, 2% acetonitrile in water; *B*=0.1% formic acid in acetonitrile) at 300 nl min^−1^ was used to elute peptides from the Pepmap capillary (0.075 × 150 mm) reverse-phase column. Spectra were recorded with the Xcalibur 2.0.7 software (Thermo Fischer scientific). Spectral data were analysed using the MaxQuant 1.3.0.5 software. Databases used were: CPS_mouse_2012_10 and human with the following modifications: Oxidation (M), Carbamidomethylation (C), SILAC modifications and Phosphorylation (STY).

### Statistics

Most results are presented as the mean±s.e.m. Variance analyses were systematically carried out for three or more sample conditions and mean values were compared pair-to-pair with the Student's *t*-test using the Prism software (GraphPad Software, La Jolla, USA). For most experiments, significance threshold was fixed at *P* value≤0.05. For endosomal tracking experiments, as the values were not normally distributed (Shapiro–Wilk test), the Kruskall–Wallis test was used for variance analysis and the Mann–Whitney test for pair-to-pair comparisons. For the Kaplan–Meier curve representing the overall survival of injected mice, statistical analysis was performed using the Log-rank (Mantel–Cox) test. For Array-CGH relapse-free analysis, statistical analyses were performed with the EpiInfo 6.04 software package from the Center for Disease Control and Prevention (Atlanta, USA) for classical *χ*^2^-tests and with the Stata 9.0 software package (StataCorp LP, College Station, USA) for survival analysis. Relapse-free survival was defined as the time from surgery to the first local or distant relapse. Contralateral tumours were also considered as a recurrence. For immunohistochemical analysis, associations between the expression of TOM1L1 and ER or ERBB2 were investigated using the Fischer's test.

## Additional information

**How to cite this article:** Chevalier, C. *et al.* TOM1L1 drives membrane delivery of MT1-MMP to promote ERBB2-induced breast cancer cell invasion. *Nat. Commun.* 7:10765 doi: 10.1038/ncomms10765 (2016).

## Supplementary Material

Supplementary Information Supplementary Figures 1-10 and Supplementary Tables 1-5.

Supplementary Movie 1 Dynamic of mCherry-MT1-MMP in sh-Ctrl, sh-TOM1L1 or rescued BT-474 cells. Live timelapse imaging of confocal orthogonal projections (X/Z) of BT-474 cells that express the indicated shRNAs and were transfected with mCherry-MT1-MMP and GFP or GFP-TOM1L1 or GFP-δGAT. Cells were plated on Oregon-Green 488-Gelatin coated glass bottom dishes. Note the global dynamic and appearance of MT1-MMP at the basal plasma membrane in control cells but not in TOM1L1-depleted cells. This dynamic is rescued by GFP-TOM1L1 but not by GFP-δGAT (Upper panel: filled arrows), correlating with gelatin degradation ability of the cells visualized with classical confocal imaging (X/Y) (Lower panel: outlined arrows). Dotted-lines show Z section used for orthogonal imaging. Movies were captured using a Leica 63x/1.4 Oil HCX PL APO CSCS objective on confocal microscope Leica SP5-SMD. Images were taken every 1.5 s during 1 min. Images were treated using 0.5 pixel-Median-Filter (ImageJ).

Supplementary Movie 2 MT1-MMP pHluorin exocytosis in SKBR3 cells. SKBR3 infected with indicated virus were transfected with MT1-MMP pHluorin and plated on plasma clean glass coverslips coated with gelatin for 3h and imaged in DMEM 1% FBS containing 25mM HEPES using a homemade TIRF setup as described for movie 3. Note that TOM1L1 increases the number of high intensity bursts that were more stable than in 3T3-neu

Supplementary Movie 3 MT1-MMP pHluorin exocytosis in 3T3-neu cells. 3T3-neu cells infected with indicated virus were transfected with MT1-MMP pHluorin and plated on plasma clean glass coverslips coated with gelatin for 3h and imaged in DMEM 1% FBS containing 25mM HEPES using a homemade TIRF setup based on a Zeiss Axiovert 200 inverted microscope, equipped with an alpha Plan-Fluar 100x/1.45 NA objective. Images were taken each 100ms for 40 s. Note that TOM1L1 increase the number of high intensity bursts.

Supplementary Movie 4 mCherry-MT1-MMP trafficking in 3T3-neu cells. Live time-lapse imaging of mCherry-MT1-MMP transfected in 3T3-neu cells infected with the indicated viruses and plated on native gelatin matrix. Note that the mCherry-MT1-MMP trafficking is strongly increased in TOM1L1-expressing cells and that mCherry-MT1- MMP-tagged endosomes are put on tracks by TOM1L1. Movies were captured using a Nikon 100X PL APO VC 1.4 oil objective on an inverted Nikon TE Eclipse microscope. Images were taken every 230 ms for 1 min. Graphs show movements of randomly selected endosomes.

Supplementary Movie 5 mCherry-MT1-MMP and GFP-RAB7 co-trafficking in 3T3-neu cells expressing TOM1L1 and its regulation by Taxol. Live time-lapse imaging of mCherry-MT1-MMP and GFP-RAB7 transfected in 3T3- neu cells that express TOM1L1. Cells were plated on native gelatin matrix and treated with 2 μM Paclitaxel to stabilize microtubules for the indicated time. Note the progressive paralysis of vesicles upon treatment. Movies were captured using a Leica 63x/1.4 Oil HCX PL APO CSCS objective on a Leica SP5-SMD confocal microscope. Images were taken every 390 ms for 1 min

Supplementary Movie 6 Specific regulation of mCherry-MT1-MMP/GFP-Rab7- endosomes trafficking by TOM1L1 in 3T3-neu cells. Left panel: Live time-lapse imaging of mCherry-MT1-MMP and GFP-RAB7 transfected in 3T3- neu cells infected as indicated. Cells were plated on native gelatin matrix. Note the strong effect of TOM1L1 on the long-range trafficking of MT1-MMP/Rab7 endosomes. Movies were captured using a Leica 63x/1.4 Oil HCX PL APO CSCS objective on a Leica SP5-SMD confocal microscope. Images were taken every 830 ms for 1 min. Right panel: Live time-lapse imaging of lysosomes visualized using Lysotracker-Red technology in 3T3- neu cells infected as indicated. Note that TOM1L1 or GAT deletion mutant have strictly no effect on lysosomes trafficking. Movies were captured using a Leica 63x/1.4 Oil HCX PL APO CSCS objective on a Leica SP5- SMD confocal microscope. Images were taken every 830 ms for 1 min.
