# Supplementary material for: TOM1L1 drives membrane delivery of MT1-MMP to promote ERBB2-induced breast cancer cell invasion
Source: Nat Commun. 2016 Feb 22;7:10765. doi: 10.1038/ncomms10765 (PMC4764922; doi:10.1038/ncomms10765)
Supplement: Supplementary Information — Supplementary Figures 1-10 and Supplementary Tables 1-5. [file ncomms10765-s1.pdf]

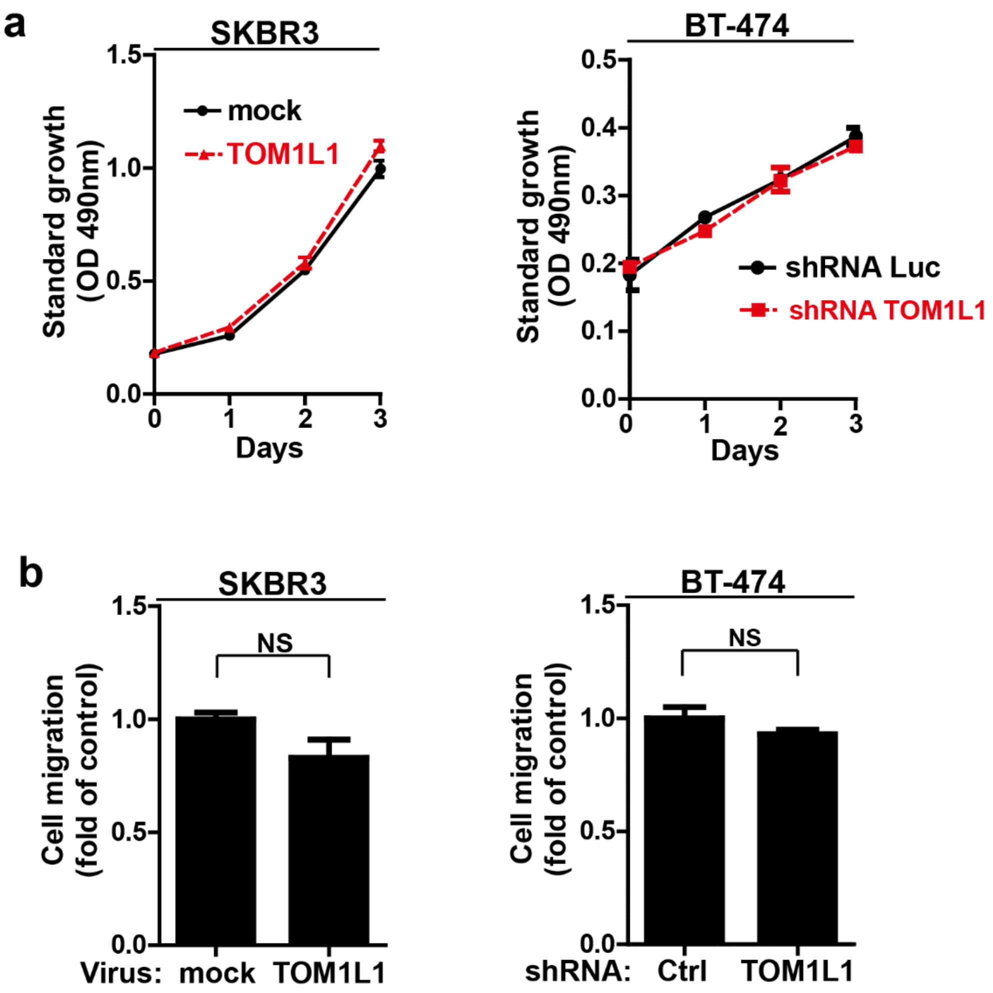


**Supplementary Figure 1: TOM1L1 does not regulate cell proliferation nor migration. (a)** Standard proliferation curves of SKBR3 and BT-474 cells infected with the indicated viruses. **(b)** Boyden chamber migration assay. 20000 SKBR3, BT-474 or 3T3-neu cells infected with the indicated viruses were seeded in the upper chamber. After 45 min, cells present in the lower chamber were counted in whole well. Histograms (mean ± SEM) show the cell migration normalized to the control condition (n=3). NS = no-significant (Student's t-test).

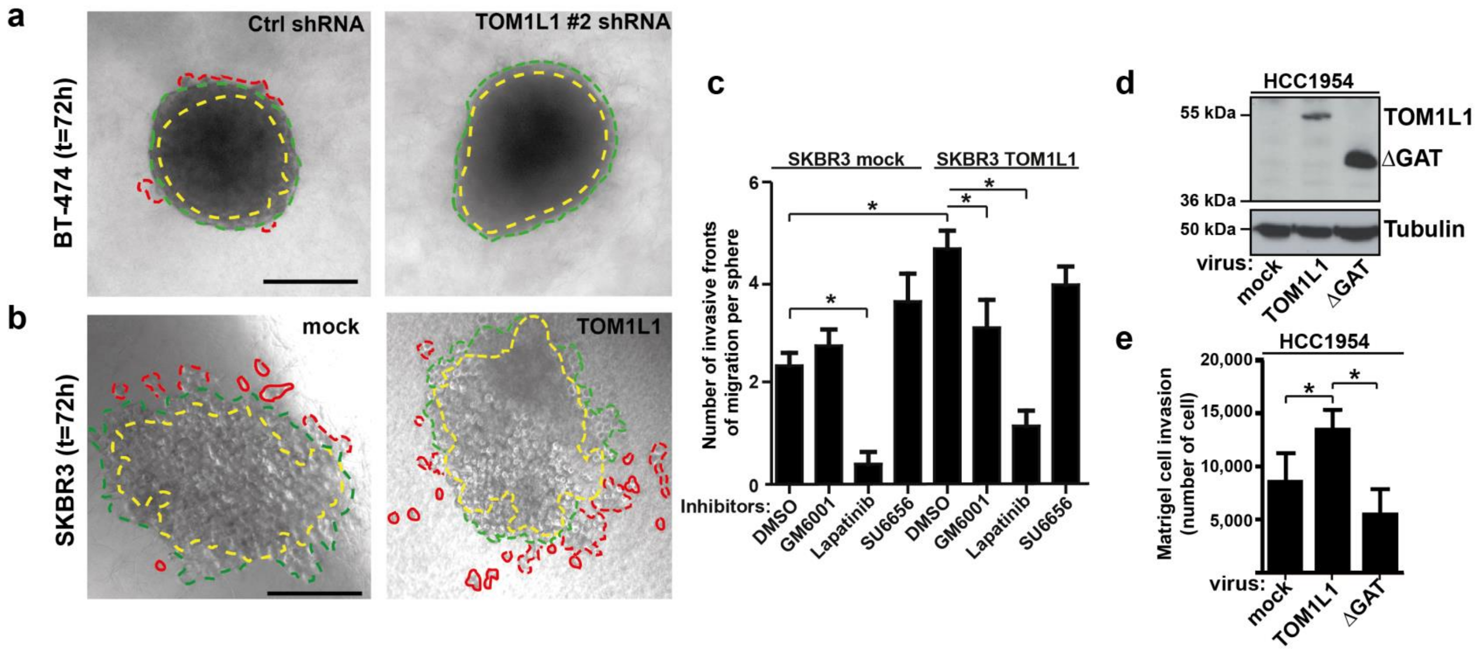


**Supplementary Figure 2: TOM1L1 regulates 3D spheroids invasion in rat tail collagen and HCC1954 invasion via its GAT domain. (a-b)** Phase contrast images of muticellular spheroids from BT474 **(a)** and SKBR3 **(b)** cells infected with the indicated virus or shRNA and embedded in telopeptides intact rat tail collagen I for 72h. Scale bar: 100µm. Yellow dotted lines represent size of spheroids at t= 0h. Green dotted lines represent sphere size at the end of the experiment and red dotted line show invasive fronts or cells. **(c)** Multicellular spheroids from SKBR3 infected as indicated were embedded in a matrix made of a mixture of bovine collagen1 and Matrigel as described in Fig. 2 and incubated for 72h in the presence of DMSO, 12.5µm GM6001, 1µM Lapatinib, 2µM SU6656. Invasive fronts emanating from spheroids were counted (n=4). Shown is the mean ± SEM *p<0.05 (Student's t-test). **(d)** HCC1954 cells were infected with the indicated virus and immunoblotted for TOM1L1 or ΔGAT expression. **(e)** Invasion of HCC1954 cells was assessed onto Boyden chambers coated with Matrigel for 48h and the number of cells in the lower chamber was counted. Shown is the mean ± SEM (n=3). *p<0.05 (Student's t-test).

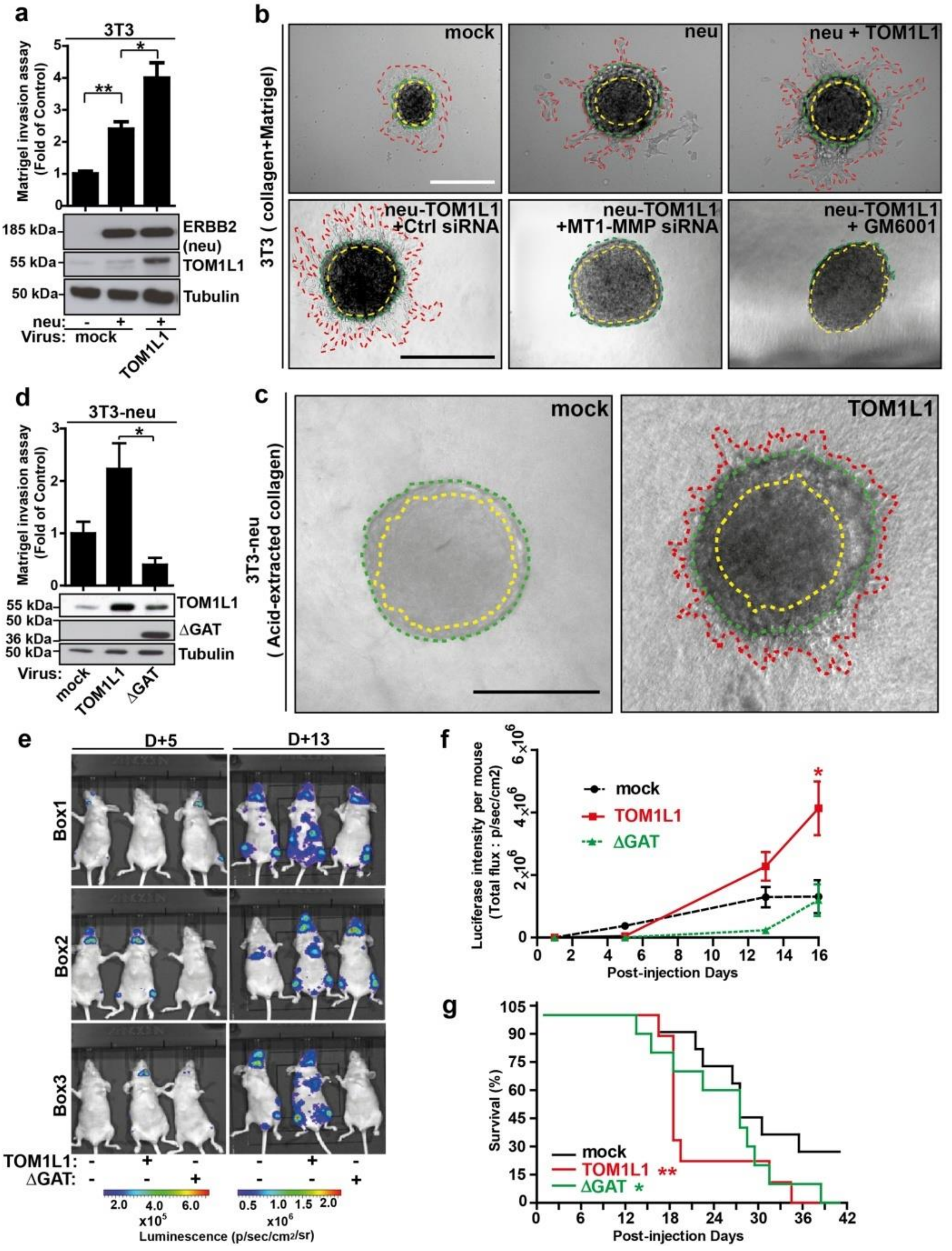
a
3T3
Matrigel invasion assay (Fold of Control)
ERBB2 (neu)
TOM1L1
Tubulin
185 kDa
55 kDa
50 kDa
neu:
Virus: mock
TOM1L1
b
3T3 ( collagen+Matrigel)
mock
neu
neu + TOM1L1
neu-TOM1L1 +Ctrl siRNA
neu-TOM1L1 +MT1-MMP siRNA
neu-TOM1L1 + GM6001
d
3T3-neu
Matrigel invasion assay (Fold of Control)
TOM1L1
ΔGAT
Tubulin
55 kDa
50 kDa
36 kDa
Virus: mock TOM1L1 ΔGAT
c
3T3-neu ( Acid-extracted collagen)
mock
TOM1L1
e
D+5
D+13
Box1
Box2
Box3
TOM1L1:
ΔGAT:
x10^5
x10^6
Luminescence (p/sec/cm2/sr)
f
Luciferase intensity per mouse (Total flux : p/sec/cm2)
mock
TOM1L1
ΔGAT
Post-injection Days
g
Survival (%)
mock
TOM1L1 **
ΔGAT *
Post-injection Days

**Supplementary Figure 3: TOM1L1 regulates 3T3-neu cell invasion via GAT domain in vitro and in vivo. (a)** Lower panel: whole cell lysate of 3T3-neu cells infected and immunoblotted as indicated. Upper panel: invasion of 3T3-neu cells infected with the indicated viruses was assessed in Boyden chamber with Matrigel. Mean ± SEM *: $p \leq 0.05$ (Student's t-test). **(b)** 3D spheroid invasion assay. 3T3 cells expressing neu and/or TOM1L1 and transfected with indicated siRNAs or treated with 12.5µM of GM6001 for 3h were plated in a 3D collagen/Matrigel matrix. Invasion was observed by timelapse microscopy for 30 to 48 h. **(c)** Same as in (b) but in telopeptides intact rat tail collagen I and after 72h incubation. Yellow dotted lines represent size of spheroids at t= 0h. Green dotted lines represent their size at the end of the experiment and red dotted line show invasive strands. Invasive strands are present in both matrix conditions. Scale bars: 150µm. **(d)** Role of the GAT domain. Top panel: 3T3-neu cells were infected with indicated viruses and were seeded on Boyden chambers coated with Matrigel. Cell invasion was measured as in (a). Bottom panel: western blot showing expression of TOM1L1 and ΔGAT mutant in indicated cells. **(e)** In vivo invasion assay. One million luciferase-expressing 3T3-neu cells infected with the indicated viruses were injected in the heart left ventricle. 48h later, metastasis formation was imaged in whole body after luciferin injection. Representative images of 3 mice per condition at day 5 and 13 (D+5, D+13) after injection are shown. The color code is indicated in the scale under the images. **(f)** Quantification of luciferase intensity in time per mouse (mock: n = 5, TOM1L1: n = 5, ΔGAT: n = 5). *: $p \leq 0.05$ (Student's t-test). **(g)** Kaplan-Meier survival analysis of mice after injection of 3T3-neu cells (mock: n = 11, TOM1L1: n = 9, ΔGAT: n = 10). *: $p \leq 0.05$ (Log-rank, Mantel-Cox test).

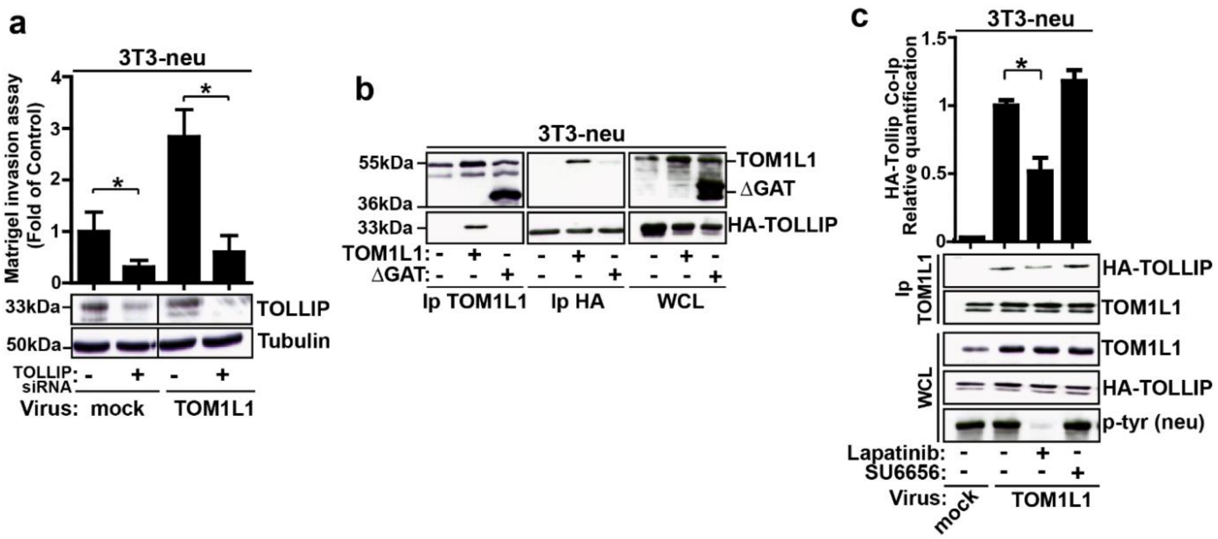


**Supplementary Figure 4: TOM1L1 regulates 3T3-neu cell invasion via GAT association with Tollip.** **(a)** Lower panel: 3T3-neu cells infected as indicated were immunoblotted after transfection of anti-mouse Tollip or control siRNAs. Upper panel: 3T3-neu cells infected and transfected as in lower panel were assessed for invasion in Boyden chambers with Matrigel. *: $p \leq 0.05$ (Student's t-test). **(b)** 3T3-neu cells infected as indicated were transfected with HA-tagged TOLLIP. 48 h after transfection, whole cell-lysates (WCL) were immunoprecipitated (Ip) and immunoblotted as indicated. **(c)** 3T3-neu cells infected as indicated were transfected with HA-TOLLIP. 48 h after transfection cells were treated with 1 µM Lapatinib or 5 µM SU6656 for 3 h. Lysates were immunoprecipitated and immunoblotted as indicated. Upper graph represents the relative quantification of HA-TOLLIP/TOM1L1 co-immunoprecipitation in 3 independent experiments (n=3). Mean ± SEM. *: $p \leq 0.05$ (Student's t-test).

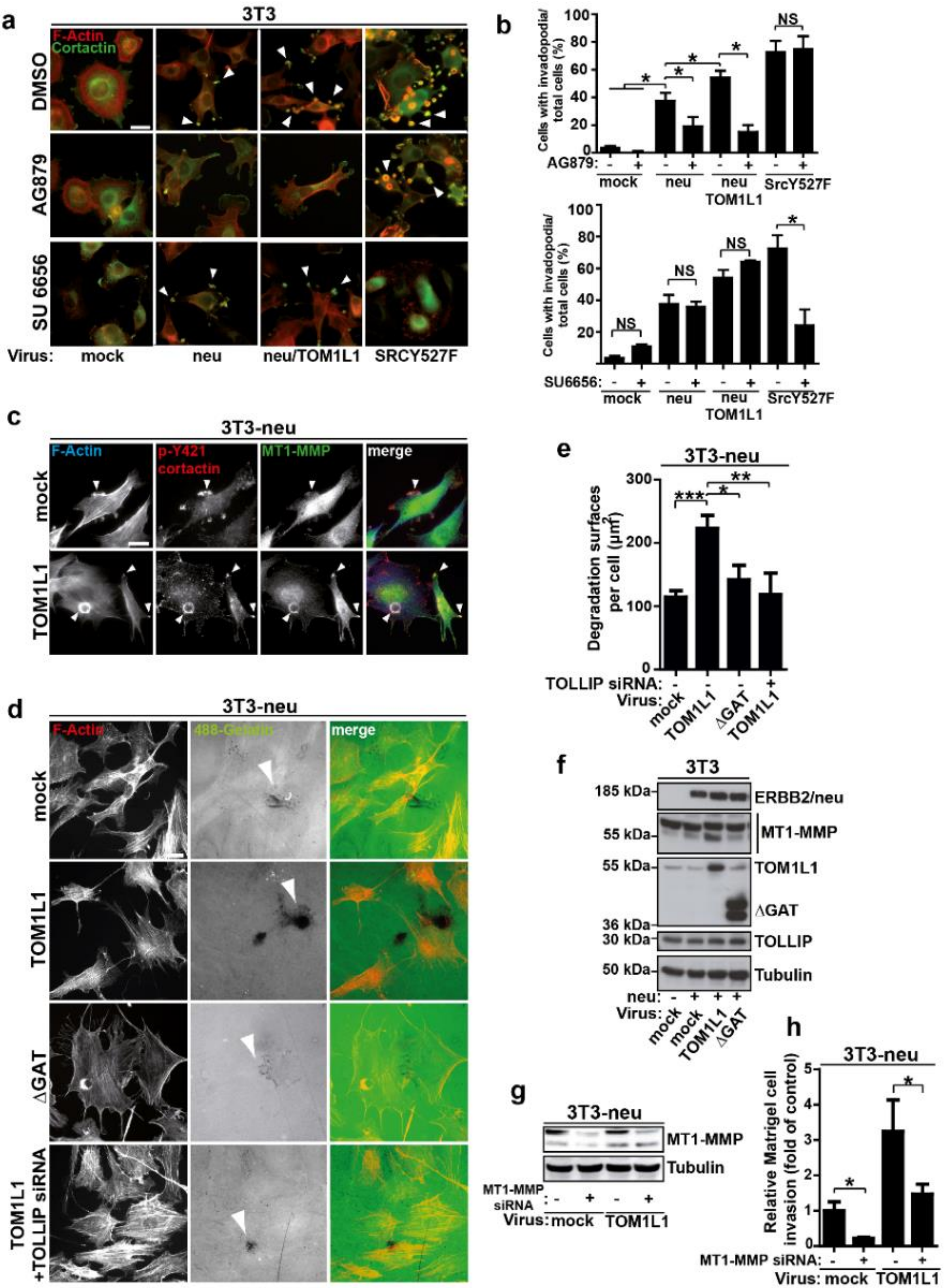

a
3T3
F-Actin
Cortactin
DMSO
AG879
SU 6656
Virus: mock neu neu/TOM1L1 SRCY527F
b
Cells with invadopodia/ total cells (%)
AG879:
mock
neu
neu TOM1L1
SrcY527F
NS
SU6656:
c
3T3-neu
F-Actin
p-Y421 cortactin
MT1-MMP
merge
mock
TOM1L1
d
3T3-neu
F-Actin
488-Gelatin
merge
mock
TOM1L1
ΔGAT
TOM1L1 +TOLLIP siRNA
e
3T3-neu
Degradation surfaces per cell (µm2)
TOLLIP siRNA:
Virus: mock TOM1L1 ΔGAT TOM1L1
f
3T3
185 kDa
55 kDa
36 kDa
30 kDa
50 kDa
ERBB2/neu
MT1-MMP
TOM1L1
ΔGAT
TOLLIP
Tubulin
neu:
Virus: mock mock TOM1L1 ΔGAT
g
3T3-neu
MT1-MMP
Tubulin
MT1-MMP siRNA:
Virus: mock TOM1L1
h
3T3-neu
Relative Matrigel cell invasion (fold of control)
MT1-MMP siRNA:
Virus: mock TOM1L1

**Supplementary Figure 5: The ERBB2-induced TOM1L1-TOLLIP complex regulates invadopodia formation and MT1-MMP activity in 3T3-neu cells. (a)** Representative wide-field images showing invadopodia (arrowheads). 3T3 cells infected with the indicated viruses were seeded on Matrigel-coated coverslips with or without 1 µM AG879 for 3 hours. Cells were stained with Alexa-592 phalloidin for F-Actin detection and immunolabeled for Cortactin. Scale bar: 20µm. **(b)** Quantification of (a): cells with invadopodia were counted and the results normalized to the total number of cells/field (%, n=30-50 cells). *: $p \leq 0.05$; NS: non-significant (Student's t-test). **(c)** Representative confocal images of F-Actin, p-Y421 Cortactin and MT1-MMP in 3T3-neu cells infected with the indicated viruses. Cells were seeded on gelatin-coated coverslips for 6h at 37°C and immunolabeled using specific antibodies. Arrowheads show colocalization spots. Scale bar: 20µm. **(d)** In situ zymography assays. 3T3-neu cells infected with the indicated viruses and transfected or not with TOLLIP siRNA were seeded on Oregon-Green-488 gelatin-coated coverslips overnight at 37°C to visualize proteolytic activity of the cells (arrowheads). Cells were stained with Alexa-594 phalloidin for F-Actin detection. Scale bar: 20µm. **(e)** Quantification of (d). Histograms represent mean ± SEM of the degradation surface observed per cell. Mock: n= 87, TOM1L1: n=61, ΔGAT: n=62, TOM1L1 with TOLLIP siRNA: n=22. *: $p \leq 0.05$; **: $p \leq 0.01$; ***: $p \leq 0.001$(Student's t-test). **(f)** Western blotting of ERBB2, MT1-MMP, TOM1L1, GAT deletion mutant (ΔGAT) and TOLLIP expression in 3T3-neu cells. Note the apparition of active (55 kDa) isoform of MT1-MMP when TOM1L1 is expressed. **(g)** 48h after control or MT1-MMP siRNA transfection, whole cell lysates of 3T3-neu cells infected as indicated were immunoblotted for detection of MT1-MMP. **(h)** 48h after control or MT1-MMP siRNA transfection, invasion of 3T3-neu cells infected with the indicated viruses was evaluated in Boyden chambers with Matrigel.

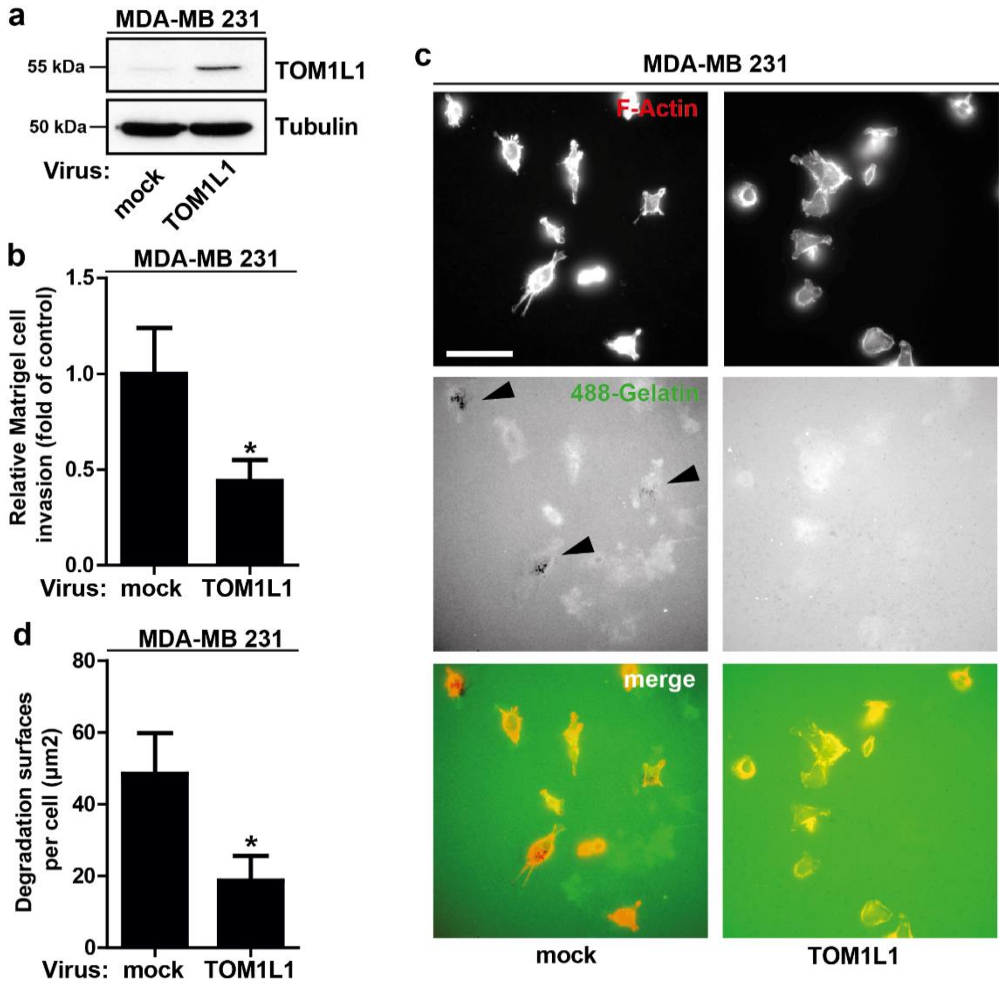


**Supplementary Figure 6: TOM1L1 inhibits invasive properties and invadopodia activity of MDA-MB 231 cells. (a)** Western blot showing TOM1L1 expression in MDA-MB231 cells infected with the indicated viruses. **(b)** MDA-MB 231 cells infected with the indicated viruses were assessed for invasion in Boyden chamber with Matrigel. Histograms (mean ± SEM) show the cell migration normalized to the control condition. (n=3) *: $p \leq 0.05$ (Student's t-test). **(c)** MDA-MB 231 cells infected with the indicated viruses were seeded on Oregon-Green 488-Gelatin coated coverslips and incubated 3h at 37°C to visualize proteolytic activity of the cells (arrowheads). Cells were stained with Alexa-594 phalloidin for F-Actin detection. Scale bar: 20µm. **(d)** Quantification of the matrix degradation area per cell (n=20). Mean ± SEM *: $p \leq 0.05$ (Student's t-test).

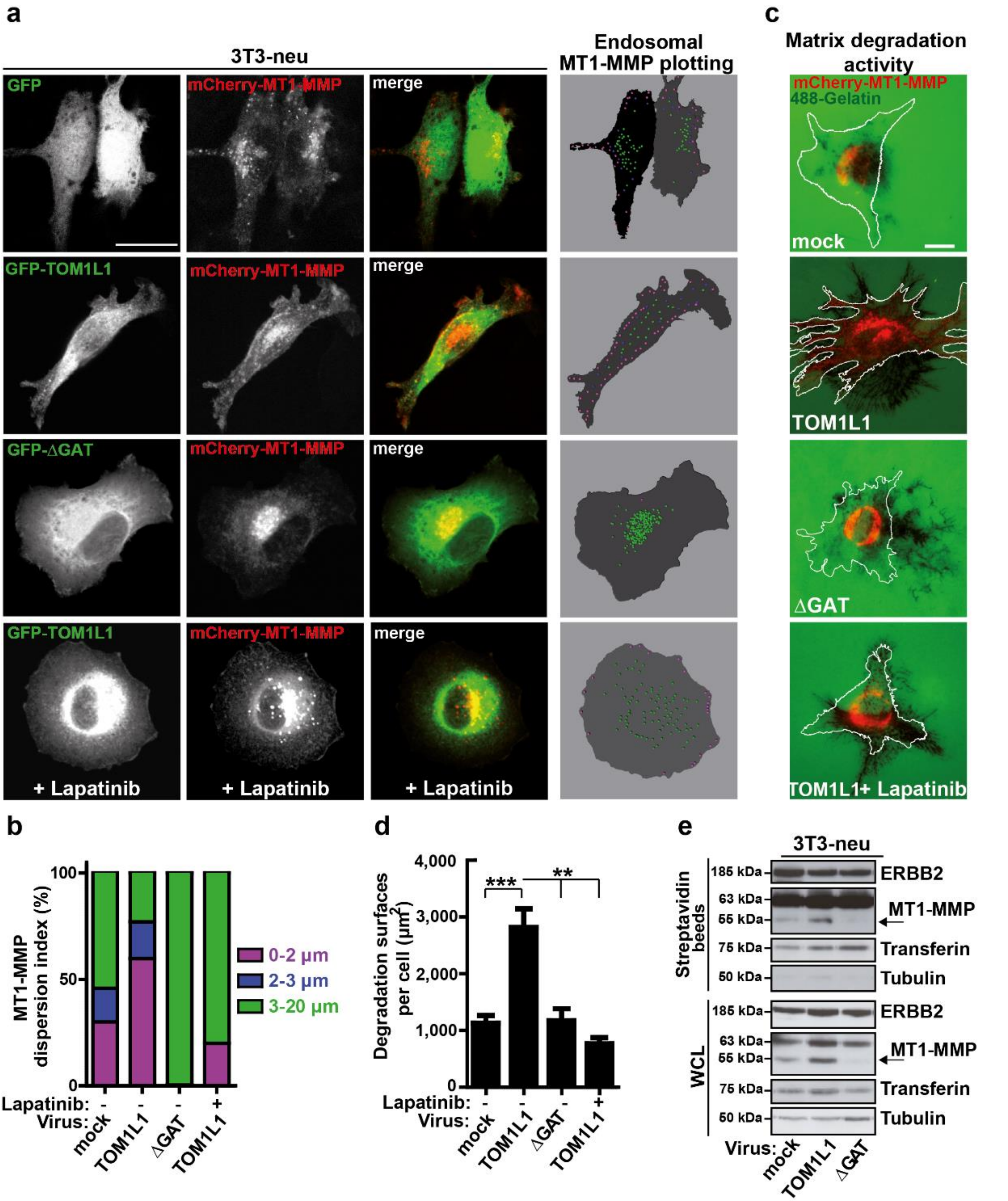
a
3T3-neu
Endosomal MT1-MMP plotting
GFP
mCherry-MT1-MMP
merge
GFP-TOM1L1
GFP-ΔGAT
+ Lapatinib
c
Matrix degradation activity
488-Gelatin
mock
TOM1L1
ΔGAT
TOM1L1+ Lapatinib
b
MT1-MMP dispersion index (%)
100
50
0
0-2 µm
2-3 µm
3-20 µm
Lapatinib:
Virus:
mock
TOM1L1
ΔGAT
d
Degradation surfaces per cell (µm2)
4,000
3,000
2,000
1,000
***
**
e
185 kDa
63 kDa
55 kDa
75 kDa
50 kDa
ERBB2
MT1-MMP
Transferin
Tubulin
Streptavidin beads
WCL

**Supplementary Figure 7: TOM1L1 promotes membrane partitioning of MT1-MMP for invadopodia activity and ECM degradation. (a)** Left Panel: Representative confocal images showing localization of mCherry-MT1-MMP, GFP-TOM1L1 and GFP-ΔGAT mutant. 3T3-neu cells were transfected with mCherry-MT1-MMP and GFP, GFP-TOM1L1 or GFP-ΔGAT and then plated on gelatin-coated coverslips for 3h treated or not with 1µM Lapatinib. Scale bar, 20 µm. Rigth panel: Endosomal MT1-MMP plotting. Images were analyzed using the Imaris software and the distribution of the endosomes as a function of their distance from the cell border was quantified. Endosomes located near the border contact are in purple (0-2µm), more cytosolic endosomes (2-3µm) are in blue and endosomes far from the border are in green. **(b)** Quantification of MT1-MMP dispersion. **(c)** 48h after mCherry-MT1-MMP transfection, 3T3-neu cells infected with the indicated viruses were seeded on Oregon-Green-488 gelatin-coated coverslips and treated or not with 1µM Lapatinib for 3h to visualize gelatin degradation areas. Scale bar: 10 µm. **(d)** Histograms show mean ± SEM of the quantification of 25-42 gelatin degradation areas/condition. **: $p \leq 0.01$; ***: $p \leq 0.001$ (Student's t-test). **(e)** Immunoblotting of cell surface biotinylated ERBB2, MT1-MMP and Transferin receptor after Streptavidin beads precipitation in 3T3-neu cells.  Arrows show catalytically active cleaved form of MT1-MMP.

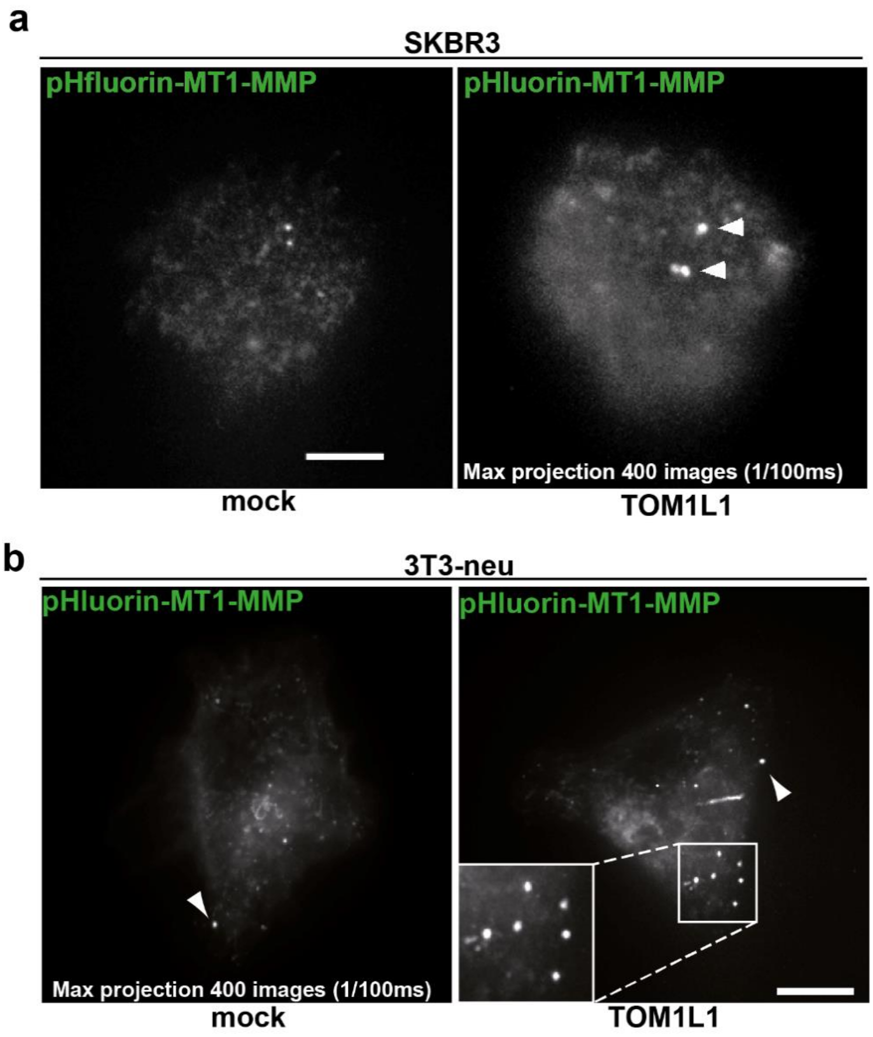


**Supplementary Figure 8: MT1-MMP exocytosis.** 48h after transfection with MT1-MMP-pHluorin construct as in ref [34], SKBR3 **(a)** or 3T3-neu cells **(b)** were plated on plasma clean glass coverslip coated with gelatin and imaged by TIRF in live (see Supplementary Movies 2 & 3). The max projection of 400 images taken every 100ms during 40s is shown. Arrowheads point exocytose flashes. Insets are higher magnification of the boxed areas showing region of high MT1-MMP exocytosis. Scale bar: 10µm.

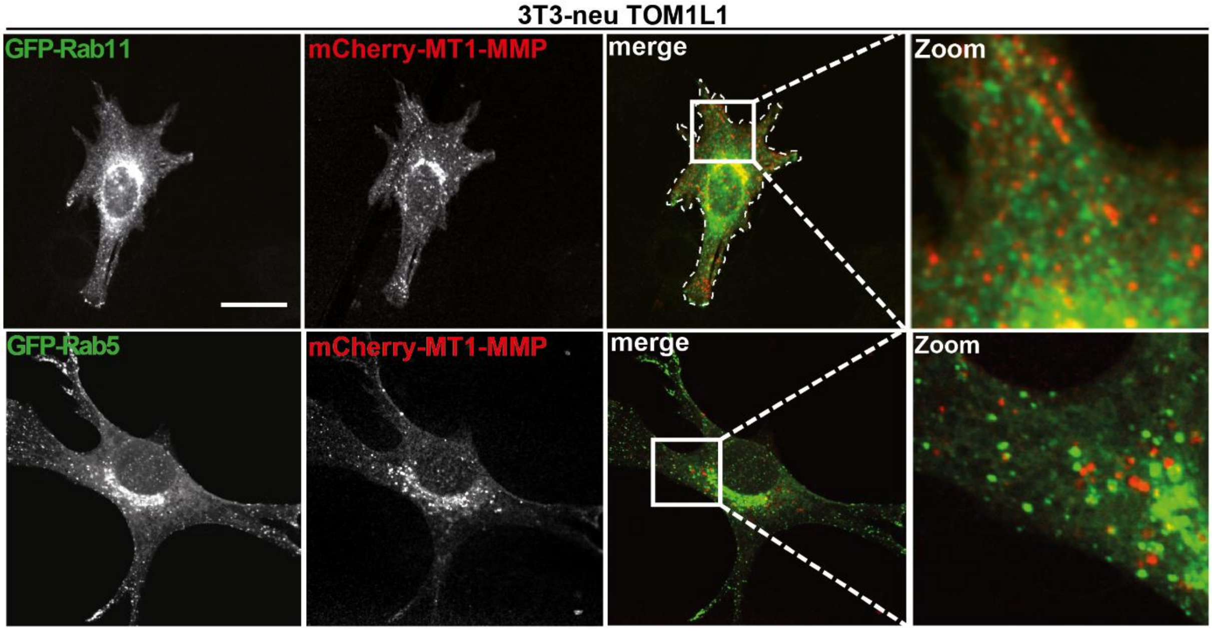


**Supplementary Figure 9: MT1-MMP is not located in RAB5/RAB11-endosomes.** 48h after GFP-Rab11 or GFP-Rab5 and mCherry-MT1-MMP transfection, 3T3-neu TOM1L1-expressing cells were plated on gelatin-coated coverslips for 3h to visualize colocalization by confocal imaging. Insets show higher magnification of the boxed areas. Scale bar: 20 µm.

**Fig. 1d**

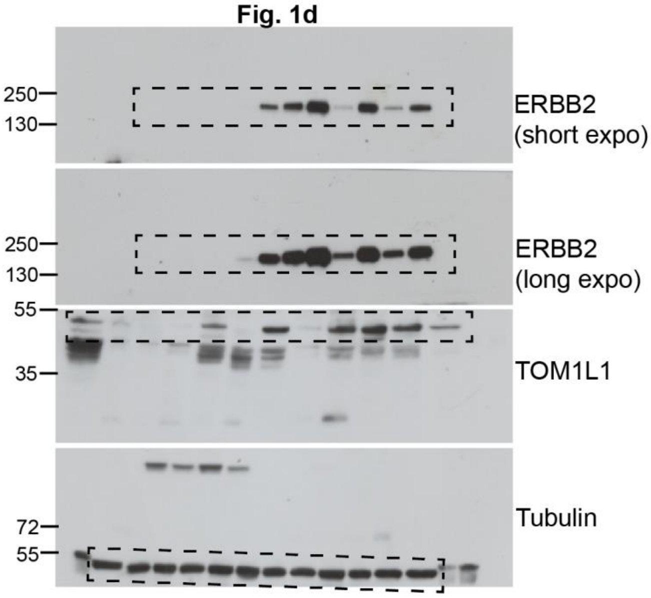


**Fig. 2a**

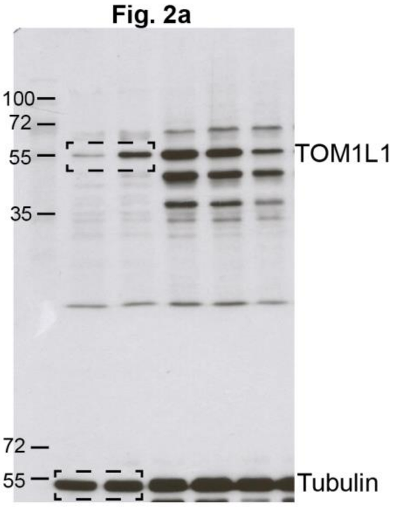


**Fig. 2e**

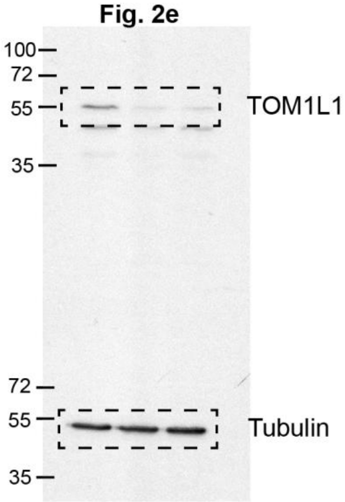


**Fig. 3b**

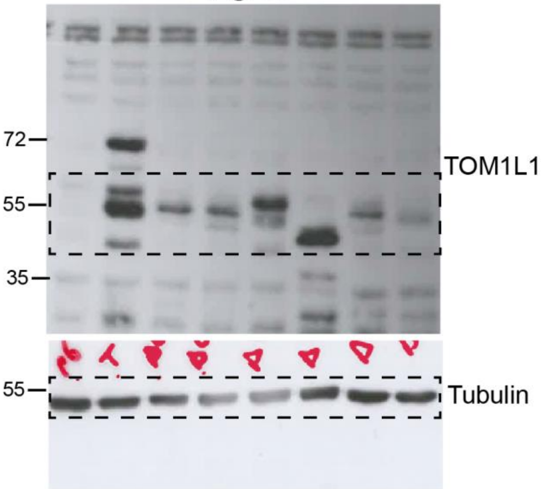


**Fig. 3c**

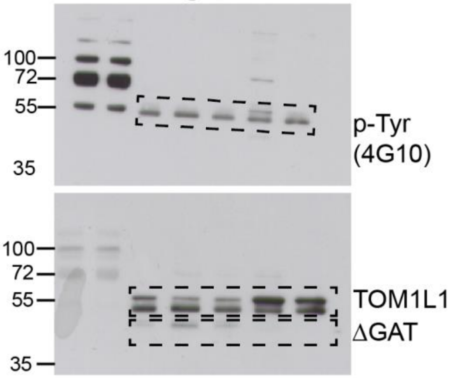


**Fig. 3f**

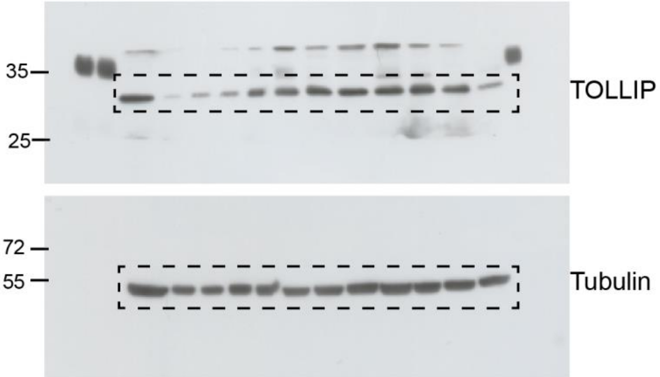


**Fig. 3g**

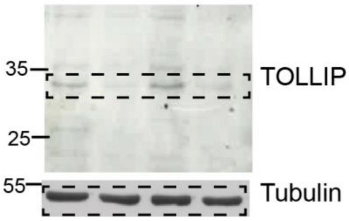


**Fig. 3h**

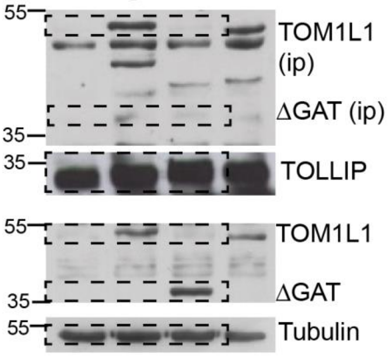

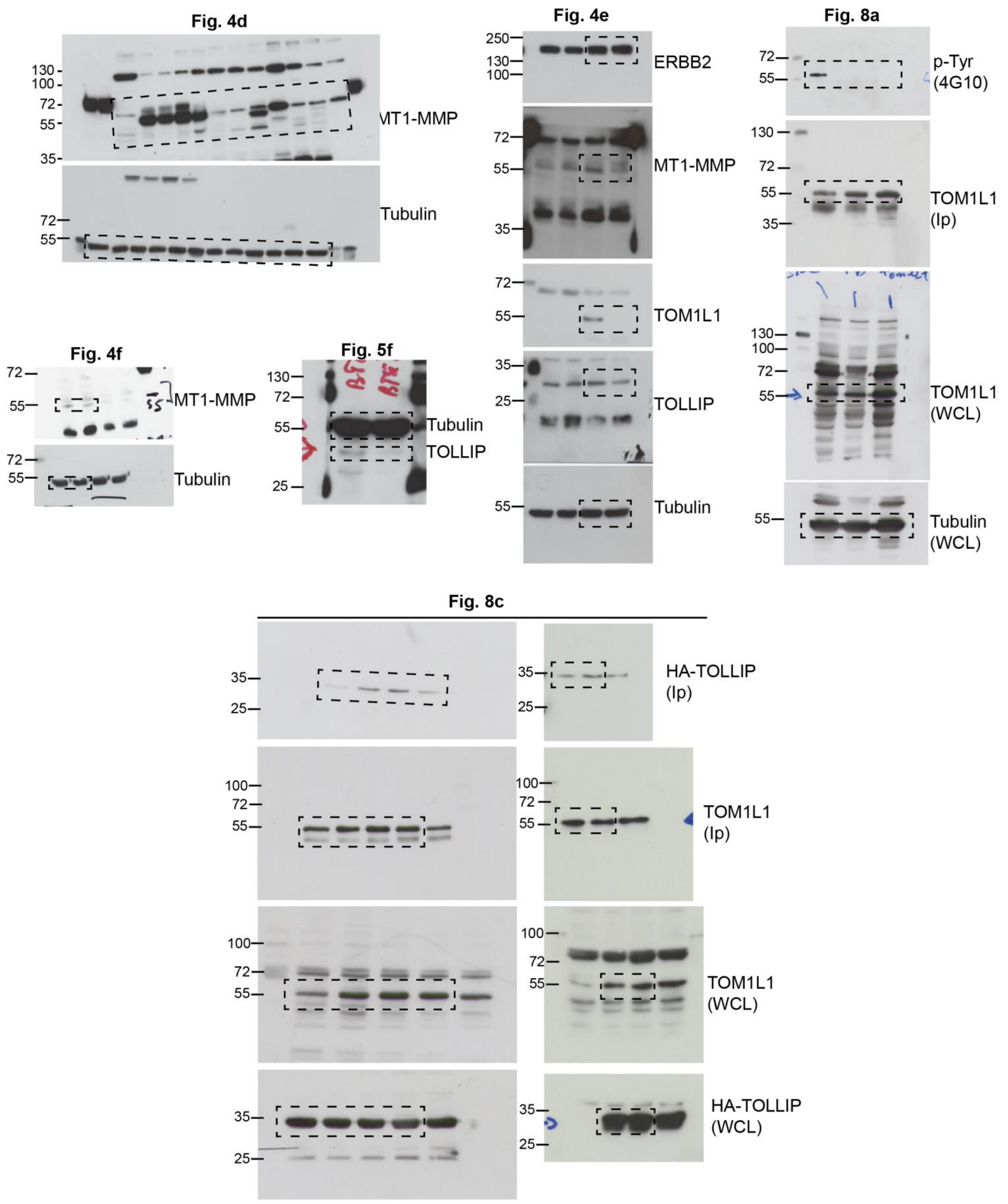


**Supplementary Figure 10: Western blot images of the selected portion displayed in the main figures.** Boxed areas were cropped for designated figures.

| Gene Symbol | Cytoband | Gene amplification (%) | Frequency of co-amplification with ERBB2 | Bilateral t-test (p values) |
|---|---|---|---|---|
| **ERBB2** | 17q12 | 18% | NA | 0.0105 |
| **TOM1L1** | 17q22 | 11% | 52.5% | 0.0351 |
| **CLTC** | 17q23.1 | 13% | 47.5% | 0.0420 |
| **RPS6KB1** | 17q23.2 | 12% | 47.5% | 0.0161 |
| **PPM1D** | 17q23.2 | 15% | 45.4% | 0.0050 |

**Supplementary Table 1 (related to Fig. 1)** Amplification of *ERBB2* and the most frequently co-amplified genes were profiled in a set of 402 breast tumours by array-CGH. The table shows the four most frequently co-amplified genes, their chromosomal localization and frequency of co-amplification.

| A | Whole Tumour Set (N = 402) | | ER- (N = 142) | | ER+ (N = 260) | |
|---|---|---|---|---|---|---|
| | **N** | **%** | **N** | **%** | **N** | **%** |
| ***ERBB2*** | 61 | 15% | 32 | 23% | 29 | 11% |
| ***TOM1L1*** | 42 | 10% | 17 | 12% | 31 | 12% |
| ***ERBB2/TOM1L1 Co-amplification*** | 32 | 8% | 13 | 9% | 19 | 7% |

| B | ERBB2 amplified Tumours (N = 61) | | ER- (N = 32) | | ER+ (N = 29) | |
|---|---|---|---|---|---|---|
| | **N** | **%** | **N** | **%** | **N** | **%** |
| ***TOM1L1*** | 32 | 52% | 13 | 41% | 19 | 66% |
| | | | | | **p = 0.025** | |

**Supplementary Table 2 (related to Fig. 1) A**: Frequency of *ERBB2* and *TOM1L1* amplification and co-amplification in a set of 402 breast tumours analysed by RT-qPCR. Frequencies are presented for the complete set of tumours and for the Oestrogen Receptor-negative (ER-) and -positive (ER+) subsets. **B**: The frequency of *TOM1L1* amplification was assessed in the 61 *ERBB2*-amplified breast cancers of A that were classified according to their ER status. *TOM1L1* amplification was significantly more frequent in ER+ than in ER- *ERBB2* amplified tumours (Chi2 statistical test).

| | **TOM1L1 +**<br>***N*(%)** | **TOM1L1 -**<br>***N* (%)** | ***P*** |
|---|---|---|---|
| **ER +** | **17 (28%)** | **43 (72%)** | **0.027** |
| **ER -** | **4 (10%)** | **37 (90%)** | |
| **HER2 +** | **12 (44%)** | **15 (56%)** | **0.001** |
| **HER2 -** | **9 (12%)** | **65 (88%)** | |

**Supplementary Table 3 (related to Fig. 1)** The expression of TOM1L1 was assessed in 101 breast cancers and was classified according to their ER and HER2 clinical status. TOM1L1 expression was significantly more frequent in ER+ than in ER- tumours and in HER2+ than in HER2- tumours (Fisher's exact test).

| Sequence | Modifications | Mass | Charges | PEP | Score | Ratio H/L | Ratio H/L normalized | Phosphorylation Localization probability |
|---|---|---|---|---|---|---|---|---|
| DPYATSVGHLIEK | Unmodified | 1428,7249 | 2,3 | 5.913E-12 | 173,72 | 0,98 | 1,00 | |
| EATNTTSEPSAPSQDLLDLSPSPR | Unmodified | 2512,1929 | 2,3 | 1.6259E-21 | 225,74 | 0,92 | 1,01 | |
| **EATNTTSEPSAPSQDLLDLSPSPR** | **Phospho (STY)** | **2592,1592** | **2** | **2.0455E-05** | **117,03** | **1,86** | **2,05** | **EATNTTSEPSAPSQDLLDLS(0.784)PS(0.216)PR** |
| EFVKENLVK | Unmodified | 1104,6179 | 2 | 0,0076586 | 76,679 | 1,10 | 1,15 | |
| EVYLDLVK | Unmodified | 977,54335 | 2 | 8.0214E-05 | 116,37 | 1,00 | 1,02 | |
| GVQFPPSEAEAETAR | Unmodified | 1587,7529 | 2,3 | 6.0188E-16 | 121,79 | 0,95 | 1,05 | |
| KGVQFPPSEAEAETAR | Unmodified | 1715,8479 | 2,3 | 3.821E-09 | 153,94 | 0,95 | 1,02 | |
| LHSELDMVK | Unmodified | 1070,543 | 2 | 3.8465E-05 | 123,35 | 0,98 | 1,00 | |
| LHSELDMVK | Oxidation (M) | 1086,5379 | 2 | 0,013058 | 76,064 | 1,00 | 1,02 | |
| NSTVTLVPEQIGK | Unmodified | 1384,7562 | 2 | 9.6167E-09 | 156,24 | 0,98 | 1,00 | |
| QETAQISSNPPTSVPTAPALSSVIAPK | Unmodified | 2690,4127 | 2,3 | 1.1835E-08 | 141,36 | 0,95 | 0,97 | |
| SDLQPPNYYEVMEFDPLAPAVTTEAIYEEIDAHQHK | Oxidation (M) | 4175,931 | 4 | 0,0006373 | 83,633 | 0,95 | 0,97 | |
| SDLQPPNYYEVMEFDPLAPAVTTEAIYEEIDAHQHK | Unmodified | 4159,9361 | 4 | 9.992E-06 | 103,68 | 0,94 | 0,96 | |
| TWSQGFPGGVDVSEVK | Unmodified | 1691,8155 | 2,3 | 3.9388E-29 | 244,05 | 0,92 | 0,94 | |
| VMSAILMENTPGSENHEDIELLQK | Unmodified | 2697,299 | 2,3,4 | 1.0051E-27 | 238,69 | 0,90 | 0,92 | |
| VMSAILMENTPGSENHEDIELLQK | Oxidation (M) | 2713,2939 | 2,3 | 4.5541E-09 | 158,92 | 0,92 | 0,93 | |
| YNLPLDIQNR | Unmodified | 1244,6513 | 2 | 1.5118E-11 | 180,07 | 0,97 | 1,07 | |

**Supplementary Table 4 (related to Fig. 8) Mass-spectrometry:** Identified human-TOM1L1 peptides after trypsin digestion. The table shows the peptide sequences, H/L SILAC ratio (light: Lapatinib; heavy: DMSO) and localization probability of phosphorylation sites (red).

| | |
|---|---|
| TOM1L1-F | 5'-GCTTCCCAGGAGGTGTGGAT-3' |
| TOM1L1-R | 5'-TGAGGGAGGAAACTGAACGC-3' |
| ERBB2-F | 5'-CCTATGCTGCCTCTTAGACCA-3' |
| ERBB2-R | 5'-TTGCAATCTGCATACACCAG-3' |
| RPS6KB1-F | 5'-ACTTCCAATACGACAGCCGAA-3' |
| RPS6KB1-R | 5'-TTCATACGCAGGTGCTCTG-3' |
| PPM1D-F | 5'-ATTGAAAGAACCCCTCCAACAA-3' |
| PPM1D-R | 5'-GCCATTTCGTCTATGCTTCTTCAT-3' |
| CLTC-F | 5'-CAATTTTGGACGTTTGGCATC-3' |
| CLTC-R | 5'-AGCAGACCTCTTTCCATGTTCg-3' |
| DCK-F | 5'-GCCGCCACAAGACTAAGAAT-3' |
| DCK-R | 5'-CGATGTTCCCTTCGATGGAg-3' |
| ALB-F | 5'-GCTGTCATCTCTTGTGGGCTgT-3' |
| ALB-R | 5'-ACTCATGGGAGCTGCTGGTTC-3' |
| GAPDH-F | 5'-GAAGGTGAAGGTCGGAGTC-3' |
| GAPDH-R | 5'-GAAGATGGTGATGGGATTTC-3' |
| 28S-F | 5'-CGATCCATCATCCGCAATG-3' |
| 28S-R | 5'-AGCCAAGCTCAGCGCAAC-3' |

**Supplementary Table 5: Primers used for the RT-qPCR analysis** (F: forward; R: reverse)
